# Computational modelling studies of some 1,3-thiazine derivatives as anti-influenza inhibitors targeting H1N1 neuraminidase via 2D-QSAR, 3D-QSAR, molecular docking, and ADMET predictions

**DOI:** 10.1186/s43088-022-00280-6

**Published:** 2022-08-19

**Authors:** Mustapha Abdullahi, Adamu Uzairu, Gideon Adamu Shallangwa, Paul Andrew Mamza, Muhammad Tukur Ibrahim

**Affiliations:** 1 Present Address: Faculty of Physical sciences, Department of Chemistry, Ahmadu Bello University, P.M.B. 1044, Zaria, Kaduna State Federal Republic of Nigeria; 2 Faculty of Sciences, Department of Pure and Applied Chemistry, Kaduna State University, Tafawa-Balewa Way, Kaduna, Kaduna State Federal Republic of Nigeria

**Keywords:** Modeling, Binding score, Receptor, Neuraminidase, Residual interaction

## Abstract

**Background:**

Influenza virus disease remains one of the most contagious diseases that aided the deaths of many patients, especially in this COVID-19 pandemic era. Recent discoveries have shown that the high prevalence of influenza and SARS-CoV-2 coinfection can rapidly increase the death rate of patients. Hence, it became necessary to search for more potent inhibitors for influenza disease therapy. The present study utilized some computational modeling concepts such as 2D-QSAR, 3D-QSAR, molecular docking simulation, and ADMET predictions of some 1,3-thiazine derivatives as inhibitors of influenza neuraminidase (NA).

**Results:**

The 2D-QSAR modeling results showed GFA-MLR ($R_{{\text{train }}}^{2}$ = 0.9192, *Q*^2^ = 0.8767, *R*^2^_adj_ = 0.8991, RMSE = 0.0959, $R_{{{\text{test}}}}^{2}$ = 0.8943, $R_{{{\text{pred}}}}^{2}$ = 0.7745) and GFA-ANN ($R_{{\text{train }}}^{2}$ = 0.9227, *Q*^2^ = 0.9212, RMSE = 0.0940, $R_{{{\text{test}}}}^{2}$ = 0.8831, $R_{{{\text{pred}}}}^{2}$ = 0.7763) models with the computed descriptors as ATS7s, SpMax5_Bhv, nHBint6, and TDB9m for predicting the NA inhibitory activities of compounds which have passed the global criteria of accepting QSAR model. The 3D-QSAR modeling was carried out based on the comparative molecular field analysis (CoMFA) and comparative similarity indices analysis (CoMSIA). The CoMFA_ES ($R_{{\text{train }}}^{2}$ = 0.9620, *Q*^2^ = 0.643) and CoMSIA_SED ($R_{{\text{train }}}^{2}$ = 0.8770, *Q*^2^ = 0.702) models were found to also have good and reliable predicting ability. The compounds were also virtually screened based on their binding scores via molecular docking simulations with the active site of the NA (H1N1) target receptor which also confirms their resilient potency. Four potential lead compounds (4, 7, 14, and 15) with the relatively high inhibitory rate (> 50%) and docking (> − 6.3 kcal/mol) scores were identified as the possible lead candidates for in silico exploration of improved anti-influenza agents.

**Conclusion:**

The drug-likeness and ADMET predictions of the lead compounds revealed non-violation of Lipinski’s rule and good pharmacokinetic profiles as important guidelines for rational drug design. Hence, the outcome of this research set a course for the in silico design and exploration of novel NA inhibitors with improved potency.

## Background

Influenza virus disease remains one of the major health menaces affecting humans because of its high mortality and morbidity rates in recent times even with the devastating COVID-19 pandemic [1]. The COVID-19 pandemic has affected the socioeconomic and financial state of numerous countries around the world [2]. The recommendations of constant hand hygiene, face mask-wearing, social, and physical distancing made by public health officials have immensely helped in controlling the spread of COVID-19 and other diseases including the influenza virus disease [3]. The seasonal influenza rates are reported to be lower in previous years which could be due to the numerous COVID-19 precautions taken to slow down the spread of coronavirus [4]. However, researchers thought the decreased number of influenza cases was associated with the lack of testing which makes sense because patients with symptoms of respiratory infections are usually tested for COVID-19 [5]. In Europe, the epidemiology of seasonal influenza and respiratory syncytial virus (RSV) has dramatically changed during the COVID-19 pandemic. There was also a significant decrease in both influenza and bronchiolitis during usual peak seasons in Australia and New Zealand [2, 6]. It has been reported that coinfected patients with influenza and COVID-19 viruses were over fourfold more likely to be necessitated with ventilation support and 2.4 times more tendency to die. This shows the need for more influenza testing of COVID-19 patients in the hospital and further highlights the advantages of full vaccination against both influenza disease and COVID-19 [7]. Influenza has caused over 9.3–49 million illnesses in the USA each year since 2010 [8]. It is also estimated that influenza disease results in 31.4 million outpatients' visits and more than 200,000 hospitalizations each year. One of the longest flu seasons in recent years (2017–2018) was estimated that over 900,000 people were hospitalized and more than 80,000 people died [9]. In addition, 185 pediatric deaths were reported by the Centers for Disease Control (CDC) during the period; about 80% of these deaths occurred in children who had not received the vaccination. The World Health Organization (WHO) reported about 2–5 million cases of severe illness caused by the ravaging seasonal influenza virus epidemic which resulted in over 500,000 deaths globally [10]. These flu epidemics cause severe respiratory infections in children, adults, the elderly, and people with underlying health conditions [11]. Some of the factors that aggravate the infection include obesity, diabetes, rheumatic diseases, and so on. For example, the relationship between respiratory viral disease and obesity came to prominence during the 2009 swine influenza pandemic [12–14].

Influenza virus neuraminidase is an enzyme that catalyzes the obliteration of terminal sialic acid residues (sialidase) which aids in liberating new virions formed from the infected cells and circulating to infect the neighboring cells [15]. The neuraminidase (NA) inhibition can defend the host cells from being infected and prevent its proliferation [16]. Due to the highly preserved active site structure of neuraminidase [17], it has become an attractive molecular target for the exploration and development of novel anti-influenza inhibitors. Presently, Zanamivir (Relenza™), oseltamivir (Tamiflu™), zanamivir octanoate (Inavir™), and peramivir (Rapivab™) are the four approved neuraminidase inhibitors for influenza treatment [18]. Although there is a lot of concern concerning the advent of drug resistance effects resulting from the high variability of the influenza virus [15], it becomes necessary to explore more anti-influenza drugs that have more potent efficiency and binding modes with safer side effects than the currently available drugs. The trial-and-error approach applied in the development of new drugs has been seen to be very tedious, costly, and time-consuming [19], and many 1,3-thiazine analogs were reported to have a wide variety of pharmacological properties [20]. The main objective of this study was to apply some computational modeling concepts such as 2D-QSAR, 3D-QSAR, molecular docking, and ADMET predictions in identifying potential lead compounds of 1,3-thiazines that could be utilized for future in silico design and exploration of more potent analogs with improved bioactivities.

## Methods

### Dataset collection and NA inhibitory activities

Twenty-nine compounds of 1,3-thiazine derivatives as inhibitors of influenza (H1N1) neuraminidase (NA) were retrieved from the literature [20]. The NA inhibitory activities of the compounds were reported as percentage inhibition rates (P) at the initial concentration of 40 µg/mL, and the estimated activities were computed using the logit formula as shown in Eq. 1. Furthermore, 21 compounds were considered as a training set, while the remaining 8 compounds were used as the test set as presented in Table 1.

1 $${\text{Activity}} = \log \left( {\frac{P}{100 - P}} \right)$$

**Table 1:** Substitution arrangement of 1,3-thiazine derivatives along with their NA inhibitory activities

S. No.	*R*_1_	*R*_2_	Inhibition rates (%)	Activity
				
1	2-Cl-5-NO_2_	Et	39.94	0.1772
2	3-NO_2_	Et	16.21	0.7134
3	2-EtO	Et	37.18	0.2278
4	2-MeO	Et	68.08	-0.3290
5	4-Cl	Et	39.94	0.1772
6	3,4-diMeO	Et	29.65	0.3752
7	4-NO_2_	Et	60.40	-0.1833
8	4-N(Me)_2_	Et	19.86	0.6059
9	4-AcO	Et	25.62	0.4629
10	4-F	Et	37.04	0.2304
11	4-MeO	Et	10.25	0.9423
12	4-Me	Et	17.48	0.6740
13	3-Et-4AcO	Et	23.49	0.5128
14	2-NO_2_	*t*-Bu	52.30	-0.0400
15	3-NO_2_	(CH_2_)_2_OCH_3_	59.81	-0.1727
				
16	2-Cl-5-NO_2_	Et	34.37	0.2809
17	3-NO_2_	Et	9.79	0.9645
18	2-EtO	Et	19.28	0.6219
19	2-MeO	Et	23.78	0.5059
20	4-Cl	Et	14.11	0.7844
21	3,4-diMeO	Et	17.06	0.6868
22	4-N(Me)_2_	Et	21.22	0.5697
23	4-OH	Et	23.93	0.5023
24	4-F	Et	14.87	0.7578
25	4-MeO	Et	13.36	0.8119
26	4-Me	Et	8.03	1.0589
27	3-Et-4OH	Et	25.47	0.4663
28	2-NO_2_	*t*-Bu	17.66	0.6686
29	3-NO_3_	(CH_2_)_2_OCH_3_	11.98	0.8661

### QSAR studies

#### 2D-QSAR studies

##### Molecular descriptor calculations

The 2D structures of the dataset compounds were precisely drawn using ChemDraw software [21]. The structures were converted to 3D with the subsequent initial energy minimization at the molecular mechanics force fields (MMFF) level using Spartan 14 software. The minimized structures were further optimized at the density functional theory (DFT) level with B3LYP/631G** basis set in a vacuum to have a more realistic structural conformation when their respective equilibrium geometries were attained [22]. The pharmaceutical data exploration laboratory software (PaDEL-Descriptor) was utilized to calculate about 2000 descriptors from the optimized structures. These molecular descriptors are computed based on the steric potentials, electronic, potential hydrogen bonds of path length, relative ionization, and hydrophobicity properties of structures [23]. As such, 1D, 2D, and some 3D Java class descriptors were computed by retaining the 3D coordinates of the optimized structures.

##### Data pretreatment

The computed descriptors were pretreated by removing non-informative descriptors such as constant and highly inter-correlated descriptors. The constant descriptors with a default variance cutoff of 0.001 and inter-correlated descriptors with a coefficient cutoff of 0.8 were applied to remove the non-informative descriptors [24].

##### 2D-QSAR model building and statistical validation

The 2D-QSAR model was initially built using the Materials Studio software based on genetic function approximation (GFA) for feature selection of the best subset descriptors in the training set [22]. The Friedman lack-of-fit (LOF) as the fitness function of the GFA model during the evolution process was measured, while the scaled LOF smoothness parameter was set as the default of 0.5, although the LOF that is measured with Materials Studio slightly differs from the original Friedman formula as shown in Eq. 2.

2 $${\text{LOF}} = \frac{{{\text{SSE}}}}{{N\left[ {1 - \gamma \left( {\frac{ C + dp}{N}} \right)} \right]^{2} }}$$

where *C* is the number of the model terms other than the constants, *d* is the scaled smoothing parameter, *p* is the total number of descriptors as model terms excluding constants, *N* is the training set compounds, ${\upgamma }$ is the safety factor with a score of 0.99 which makes sure that the denominator must be equal to zero. The scaled smoothing parameter is related to the scaled LOF smoothness factor (${\upgamma })$ which was set at default 0.5 for a well-defined LOF measure as shown in Eq. 3.

3 $$d = \gamma \left( {\frac{{M - C_{\max } }}{{C_{\max } }}} \right)$$

In addition, the population sample was set to 10,000, the maximum generation was set to 1000, and the number of top equations was set to 1 for an effective model convergence [25]. The descriptor matrix of the built model was initially subjected to *the Y-Randomization test* as a measure to attest to the quality of the model before being exported to Molegro Data Modeller (MDM) for the development of the multi-linear regression (MLR) and the nonlinear regression model version based on artificial neural network (ANN) analysis [26]. The prediction capability of the GFA-MLR and GFA-ANN generated was assessed using internal validation metrics as follows:

i. The Pearson correlation coefficient (r): is a measure of the correlation of two variables x and y. It is mathematically defined as
  4 $$R = \frac{{\mathop \sum \nolimits_{i = 1}^{N} (x_{i} - \overline{x})\left( {y_{i} - \overline{y}} \right)}}{{\left( {N - 1} \right)\sigma_{x} \sigma_{y} }}$$
  where $\sigma_{x}$ and $\sigma_{y}$ are the standard deviations for the variables *x* and *y*. However, Pearson correlation coefficient squared (*r*^2^) is often used to describe relationships between two variables whose range of values is between 0 and 1.
ii. Adjusted *R*^2^: is a modification of the Pearson correlation coefficient that fine-tunes the number of descriptors used in the multi-linear regression model which will always be less than or equal to the Pearson correlation coefficient as defined below:
  5 $${\text{Adjusted}} \,\,R^{2} = 1 - \left( {1 - r^{2} } \right)\frac{N - 1}{{N - p - 1}}$$
  where *N* corresponds to the number of compounds in the training set as data points and p is the number of descriptors in the built model.
iii. Spearman’s rank correlation coefficient (*ρ*): is a well-ordered correlation coefficient that utilizes the data points hierarchy as a substitute for the raw data points, and it is defined as
  6 $$\rho = 1 - \frac{{6\mathop \sum \nolimits_{i = 1}^{N} d_{i}^{2} }}{{N\left( {N^{2} - 1} \right)}}$$
  where the raw data points are changed to ranks. d_i_ is the difference between the ranks of corresponding values of *x* and *y* and *N* is the number of data points.
iv. Cross-validated correlation coefficient often represented as (*Q*^2^): is a measure of predictive power of the regression model, and it is defined as
  7 $$Q^{2} = 1.0 - \frac{{\mathop \sum \nolimits_{i = 1}^{N} (x_{{{\text{pred}}, \,i}} - x_{{{\text{obs}}, \,i}} )^{2} }}{{\mathop \sum \nolimits_{i = 1}^{N} \left( {x_{{{\text{obs}},\,i}} - \overline{x}_{{{\text{obs}},\,i}} } \right)}}$$
  where $x_{{{\text{obs}}, \,i}}$ and $x_{{{\text{pred}}, \,i}}$ refer to the observed and predicted activity scores. The closer the score of *q*^2^ is to 1.0, the better the model’s predictive power.
v. Root-mean-square error (RMSE): is a good measure for evaluating the prediction performance of the model generated which is proportional to the observed mean score as defined below.
  8 $${\text{RMSE}} = \sqrt {\frac{{\mathop \sum \nolimits_{i = 1}^{N} \left( {x_{i} - \overline{x}} \right)^{2} }}{N}}$$

The reliability and the predictive performance of the models were also assessed with the relevant external validation metrics as proposed by some prominent QSAR scientists such as Alexendra Tropsha and Kunal Roy. Some of the external validation metrics include the predicted coefficient of determination for the test set $R_{{{\text{pred}}}}^{2}$, regression coefficients for the test set ($R_{{{\text{test}}}}^{2}$), delta modified square of correlation coefficient ($\Delta R_{m}^{2}$), coefficient of determination of Y-randomization $\left( {{\text{CR}}_{p}^{2} } \right)$, among others [27].

##### Model applicability domain (AD)

The model applicability domain is the theoretical chemical space of the compounds defined by the descriptors and the modeled activity in which the acceptable QSAR model can make reliable predictions [21]. Thus, the technique helps in detecting the structural and response outliers in the training and test set, respectively. Furthermore, the leverage approach was utilized to assess the chemical space of a QSAR model, and the plot of standardized residuals against leverage values (*h*) also known as the Williams plot was used to virtually screen the compounds [28]. As such, compounds with leverage scores less than the threshold (*h* < h*) and standardized residual scores within ± 3.0*σ* (standard deviation unit) are set to have fallen in the model's chemical space or applicability domain. The warning leverage (h*) is calculated using:

9 $$h^{*} = 3\frac{{\left( {d + 1} \right)}}{N}$$

where *d* is the number of descriptors in the model and *N* is the number of compounds as the training set.

#### 3D-QSAR studies

##### Molecular minimization and alignment

**Fig. 1:**
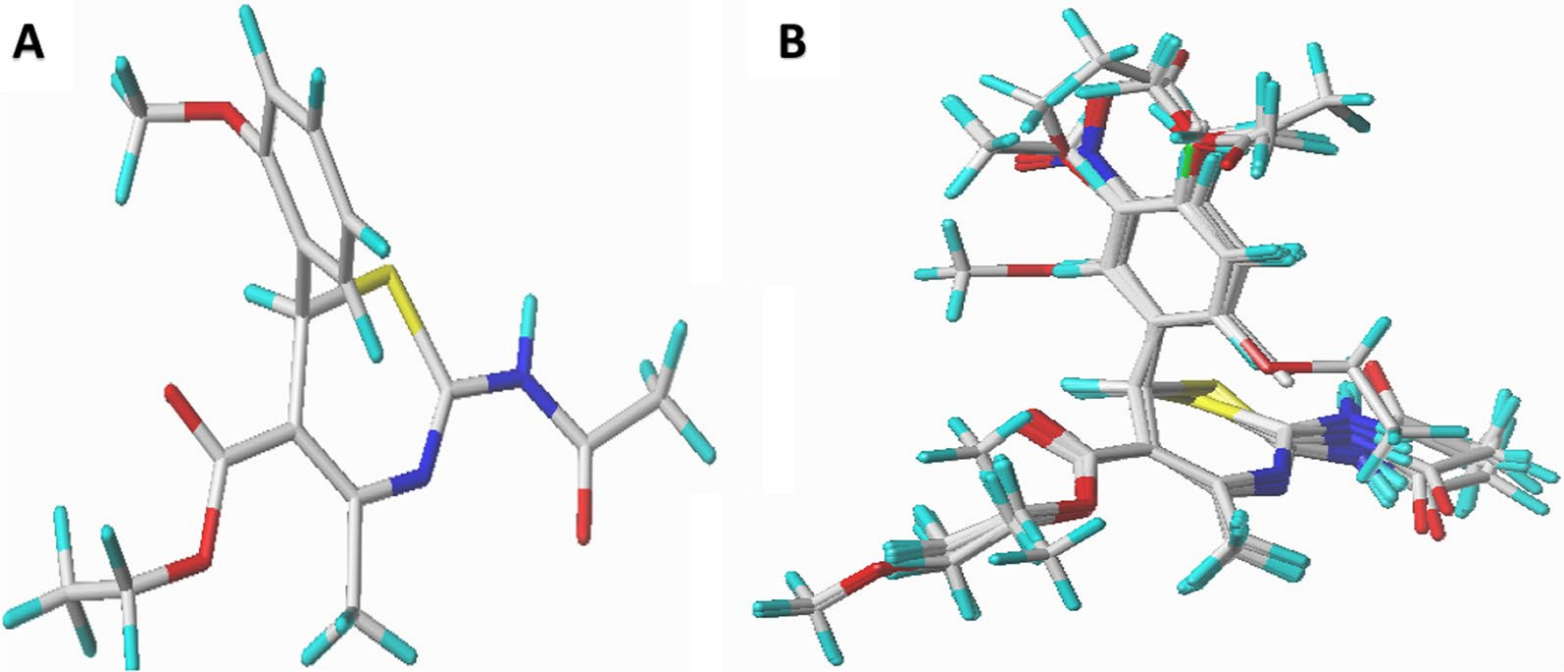
Optimized structures (**A**) structure of compound 4, (**B**) alignment and superposition of the dataset compounds (capped sticks model)

The optimized structures were minimized with Gasteiger–Huckel atomic charges of Tripos force field based on Powell conjugate gradient algorithm method at convergence criteria of 0.05 kcal/(mol Å) and 1000 maximum iterations to determine their steady conformation using Sybyl-X 2.1.1 program [29]. The molecular alignment of a database is one of the most crucial steps for building a reliable and predictive 3D-QSAR model. Hence, the distill rigid alignment was used to align the compounds in the database of the studied dataset to the most potent compound in the dataset (compound no. 4) as the template shown in Fig. 1A, B.

##### Development of 3D QSAR models

The comparative molecular field analysis (CoMFA) and comparative molecular similarity indices analysis (CoMSIA) were used for building the 3D-QSAR models [30]. The descriptor parameters utilized for the CoMFA model building were electrostatic (*E*) and steric (*S*) energies at a point in space surrounding the compounds, while the CoMSIA model was utilized for more additional descriptors such as hydrophobic (*H*), hydrogen bond donor (HBD) field, and hydrogen bond acceptor (HBA) fields [31].

##### Statistical validation of the 3D-QSAR models

The 3D-QSAR models were built by correlating the latent components from the set of available CoMFA and CoMSIA descriptors as the independent variables with the NA inhibitory activity of the compounds through partial least squares (PLS) regression analysis [32]. The competency of the 3D-QSAR models built was analyzed based on the prominent statistical validation parameters for an acceptable QSAR model.

### Molecular docking studies

Molecular docking simulation was carried out on the studied dataset using molecular operating environment (MOE) V2015.10 software. The 2009 pandemic H1N1 neuraminidase complexed with oseltamivir (PDB: 3TI6) was used as the protein receptor for the study, and the co-crystallized ligand (oseltamivir) in the receptor was used as the reference drug [33]. The best poses obtained were studied through visualization of the most stable complex formed using Discovery studio.

### Drug-likeness and ADMET prediction studies

The initial assessment of drug-likeness and pharmacokinetic parameters of potential drug candidates is key at the initial stage of the drug discovery process which aids in rolling out unfavorable effects of the candidates [34]. The pharmacokinetic parameters are based on desirable adsorption, distribution, metabolism, excretion, and toxicity (ADMET) of the query drug when administered into the body [28]. An efficient and accurate ADMETlab 2.0 Web server (https://admetmesh.scbdd.com/) was utilized to predict numerous physicochemical, drug-likeness, pharmacokinetic, and toxicity parameters of compounds in the study [35, 36]. In addition, the drug-likeness of the compounds was assessed based on Lipinski, Ghose, Veber, Egan, and Muegge rules using the SwissADME online Web server at http://www.swissadme.ch/index.php.

## Results

### 2D-QSAR modeling results

The GFA-MLR model was built using 21 compounds as a training set, while the remaining 8 compounds are the test set for the model's external validation. The GFA-MLR model with the best 4 subset descriptors is given as

11 $$\begin{aligned} {\text{Activity}} = & \, - \,0.00312656\, \times \,{\text{ATS7}}s \, + \,2.96987\, \times \,S{\text{pMax}}5\_{\text{Bhv}} \\ & - \,0.569084\, \times \,n{\text{HB}}\,\,{\text{int}}\,\,6 \, - \,0.00306718 \, \times \, {\text{TDB}}9{\text{m}} \, - \, 5.3867 \\ \end{aligned}$$

### 3D-QSAR modeling results

Before building the 3D-QSAR models, the optimized structures were automatically split based on a random method into a training set (21 compounds) and test set (8 compounds) using Sybyl-X 2.1.1 software, and the validation results were generated.

### Molecular docking results

The 1,3-thiazine derivatives (29 compounds) of the dataset were docked with the H1N1 neuraminidase receptor using MOE software as depicted earlier, and the results are shown in Table 12.

### Drug-likeness and ADMET prediction results

See Fig. 12 and Tables 15, 16, and 17.

## Discussions

### 2D-QSAR modeling studies

The 2D-QSAR modeling was performed on the 29 compounds of 1,3-thiazine derivatives as inhibitors of influenza neuraminidase. As mentioned earlier, the GFA model building protocol of Materials Studio was utilized in the feature selection of the best subset descriptors from the pool of computed molecular descriptors. It is evident from the excellent internal and external statistical parameters in Tables 2 and 3 that the model established a strong relationship between the four selected descriptors and the NA inhibitory activity. The validation metrics for the GFA-MLR model include low LOF value of 0.0546, *R*^2^ (training set) of 0.9192, adjusted *R*^2^ of 0.8991, cross-validation squared (*Q*^2^) of 0.8767, RMSE score of 0.0959, $R_{{{\text{test}}}}^{2}$ of 0.8943, and $R_{{{\text{pred}}}}^{2}$ of 0.7745 which have all passed the model criteria of accepting QSAR model. The Y-randomization test was ascertained via randomly scrambling the response activity (Y), while the model descriptors of the training set are kept constant which resulted in the construction of random models [38]. The 50 random models were generated with low *R*^2^ and *Q*^2^ scores which attested that the original model is robust and not constructed by chance [39]. The coefficient of determination for the Y-randomization test (${\text{CR}}_{p}^{2} )$ was computed as 0.8300 (≥ 0.5) which confirmed the reliability of the model generated as shown in Table 4. Hence, it was observed that all the validation criteria were fully agreed with the acceptable threshold parameters proposed [37].

**Table 2:** Internal validation of the 2D-QSAR models

Internal validation metrics	GFA-MLR model	GFA-ANN (4-5-1) model	Threshold	Comment	References
Lack of fit (LOF)	0.0546	–			
Pearson correlation (*r*)	0.9590	0.9610	*R* > 0.6	Passed	[37]
Pearson correlation squared ($R_{{\text{train }}}^{2}$)	0.9192	0.9227	*R*^2^_train_ > 0.6	Passed	[37]
Adjusted *R*^2^ (*R*^2^_adj_)	0.8991	–	*R*^2^_adj_ > 0.6	Passed	[37]
Spearman rank correlation (*ρ*)	0.9155	0.9220	*ρ* > 0.6	Passed	
Root-mean-square error (RMSE)	0.0959	0.0940	Low	Passed	
Cross-validated squared (*Q*^2^)	0.8767	0.9212	*Q*^2^ > 0.6	Passed	[37]
Y-randomization ($$cR_{p}^{2} )$$	0.8300	–	$$cR_{p}^{2}$$ > 0.6	Passed	[38]

**Table 3:** External validation parameters of the 2D-QSAR models

External validation metrics	GFA-MLR model	GFA-ANN (4-5-1) model	Threshold	Comment	References
Pearson correlation squared ($r_{{{\text{test}}}}^{2}$)	0.8943	0.8831	*R*^2^_test_ > 0.6	Passed	[37]
$R_{{{\text{pred}}}}^{2}$	0.7745	0.7763	$R_{pred}^{2}$ > 0.5	Passed	[37]
Δ$\overline{R}_{m}^{2}$(test)	0.0804	–	< 0.5	Passed	
$r_{0}^{2}$	0.8943	–	> 0.5	Passed	
RMSEP	0.1835	0.1813	–	–	
$r_{0}^{\prime 2}$	0.8923	–	> 0.5	Passed	[37]
$\left| {r_{0}^{2} - r_{0}^{\prime } } \right|$	0.0172	–	$\left| {r_{0}^{2} - r_{0}^{\prime } } \right|$< 0.3	Passed

**Table 4:** Y-randomization test of the model descriptors

Model type	*R*	*R*^2^	*Q*^2^(LOO)	Model type	*R*	*R*^2^	*Q*^2^(LOO)
Original	0.9588	0.9193	0.8767	Original	0.9588	0.9193	0.8767
Random 1	0.6840	0.4678	0.0498	Random 26	0.2884	0.0832	− 0.4710
Random 2	0.4711	0.2220	− 0.4192	Random 27	0.7986	0.6378	0.2956
Random 3	0.2190	0.0480	− 0.6641	Random 28	0.2870	0.0824	− 0.4902
Random 4	0.5558	0.3089	− 0.2649	Random 29	0.4048	0.1639	− 0.4706
Random 5	0.2217	0.0492	− 0.7146	Random 30	0.5201	0.2705	− 0.1913
Random 6	0.4871	0.2373	− 0.4865	Random 31	0.4476	0.2003	− 0.3614
Random 7	0.4123	0.1700	− 0.5249	Random 32	0.6789	0.4608	0.0217
Random 8	0.4255	0.1811	− 0.5226	Random 33	0.5206	0.2710	− 0.3008
Random 9	0.3584	0.1285	− 0.7647	Random 34	0.4066	0.1653	− 0.3498
Random 10	0.3822	0.1461	− 0.3750	Random 35	0.2158	0.0466	− 0.5317
Random 11	0.5802	0.3366	− 0.2461	Random 36	0.3436	0.1180	− 0.4491
Random 12	0.6829	0.4664	0.1653	Random 37	0.5381	0.2895	− 0.1994
Random 13	0.3048	0.0929	− 0.5982	Random 38	0.1872	0.0351	− 0.7212
Random 14	0.4534	0.2056	− 0.4894	Random 39	0.3764	0.1417	− 0.4775
Random 15	0.5593	0.3128	− 0.2535	Random 40	0.4192	0.1757	− 0.4040
Random 16	0.2342	0.0549	− 0.6505	Random 41	0.4819	0.2322	− 0.4027
Random 17	0.4352	0.1894	− 0.5397	Random 42	0.3246	0.1054	− 0.4438
Random 18	0.3545	0.1256	− 0.4689	Random 43	0.4260	0.1815	− 0.4004
Random 19	0.2646	0.0700	− 0.6156	Random 44	0.5884	0.3462	− 0.0941
Random 20	0.1861	0.0346	− 0.5696	Random 45	0.3674	0.1350	− 0.4138
Random 21	0.4574	0.2092	− 0.4509	Random 46	0.3198	0.1023	− 0.4408
Random 22	0.2850	0.0812	− 0.5281	Random 47	0.3520	0.1239	− 0.4713
Random 23	0.3162	0.1000	− 0.4651	Random 48	0.3749	0.1405	− 0.4282
Random 24	0.5506	0.3031	− 0.2223	Random 49	0.3209	0.1030	− 0.6999
Random 25	0.3291	0.1083	− 0.5492	Random 50	0.4067	0.1654	− 0.6966
Random models parameters							
		Average *R*	0.4228				
		Average *R*^2^	0.2029				
		Average *Q*^2^	− 0.3899				
		CRp^2^	0.8300				

**Fig. 2:**
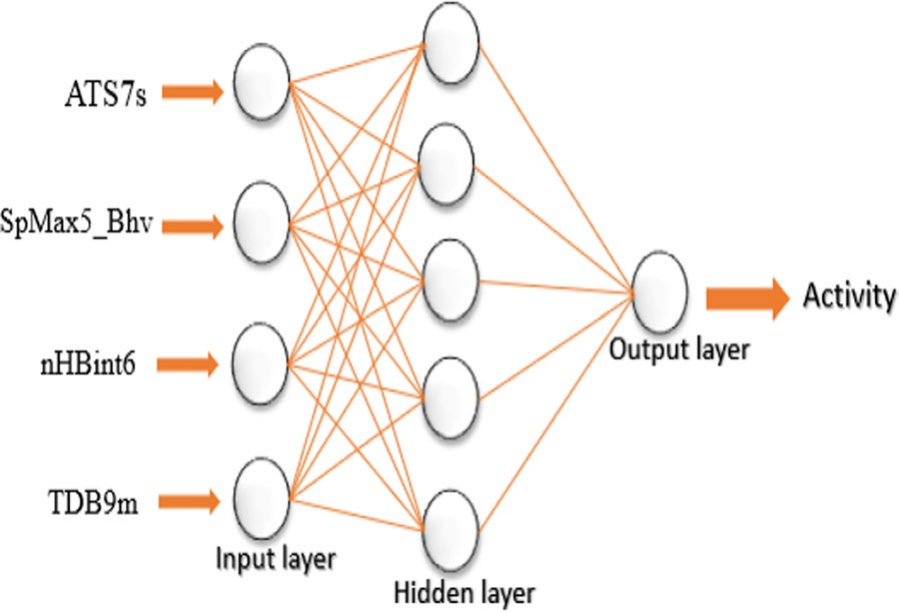
Schematic representation of the GFA-ANN (4-5-1) architecture

To explore the nonlinear effect of the model, the selected descriptors were used to construct the ANN models. The input layer consists of the four selected descriptors with a single hidden layer, and the NA inhibitory activity was used as the output layer. As such, the 4-*x*-1 ANN architecture (*x* is the number of neurons in the hidden layer) was adopted to build different ANN models, and the different value of *x* could be limited from 2 to 5 [40]. Each of the ANN model architectures was built at default settings with a maximum training epoch of 1000, the momentum of 0.2, a learning rate, and an output layer learning rate of 0.3 using the Molegro tool. For all the ANN models built, 100 epochs were sufficient to achieve stable results. The statistics of the ANN architectures are reported in Table 5, where the 4-5-1 architecture (*R*^2^ = 0.9235 and RMSE = 0.0944) was selected as the best ANN model due to its  lowest RMSE value among others. The schematic representation of the GFA-ANN (4-5-1) architecture is presented in Fig. 2. The internal and external validation results of the best ANN (4-5-1) model revealed improved statistical parameters such as R^2^ (training set) of 0.9227, cross-validation (*Q*^2^) of 0.9212, RMSE of 0.0940, $R_{{{\text{test}}}}^{2}$ of 0.8831, and $R_{{{\text{pred}}}}^{2}$ of 0.7763 as shown in Tables 2 and 3 accordingly. The description name of the model descriptors coded as ATS7s, SpMax5_Bhv, nHBint6, and TDB9m, and their numerical values which explain some essential chemical features in numerical values in predicting the anti-influenza activity are reported in Tables 6 and 7, respectively, while the parameters for the correlational analysis such as correlation coefficient, VIF, and mean effect values of the descriptors are shown in Table 8.

**Table 5:** Statistical results of different ANN model architecture

ANN architecture	*R*^2^	RMSE	RMSE_(Test)_
4-2-1	0.9163	0.0998	0.1884
4-3-1	0.9200	0.0966	0.1865
4-4-1	0.9214	0.0956	0.1851
4-5-1	0.9227	0.0940	0.1813

**Table 6:** Computed model descriptor values

Descriptor class	Descriptor code	Description
2D	ATS7s	Broto-Moreau autocorrelation—lag 7/weighted by I-state
2D	SpMax5_Bhv	Largest absolute eigenvalue of Burden modified matrix—n 5/weighted by relative van der Waals volumes
2D	nHBint6	Count of E-State descriptors of strength for potential hydrogen bonds of path length 6
3D	TDB9m	3D topological distance-based autocorrelation—lag 9/weighted by mass

**Table 7:** Computed model descriptor values

Name	ATS7s	SpMax5_Bhv	nHBint6	TDB9m	Activity	MLR Train (4D)	ANN Train (4-5-1)
*Training set*							
8	328.6200	3.1143	2.0000	355.3020	0.6059	0.6071	0.6258
9	343.3860	3.1514	2.0000	409.0160	0.4629	0.5062	0.5131
10	337.3610	2.8954	2.0000	265.4610	0.2304	0.2049	0.1982
12	317.3060	2.9889	2.0000	216.9720	0.6740	0.6943	0.7293
13	421.9410	3.1621	2.0000	356.1220	0.5128	0.4547	0.4694
14	459.1530	3.0036	2.0000	311.7580	-0.0400	0.0035	-0.0010
15	442.4260	3.0034	2.0000	358.3800	-0.1727	-0.0877	-0.0737
16	296.8580	2.9538	3.0000	149.4700	0.2809	0.2918	0.2686
17	295.9440	2.9534	2.0000	142.5230	0.9645	0.8840	0.8928
19	280.5280	2.8973	3.0000	62.4053	0.5059	0.4420	0.4644
20	203.8400	2.9016	2.0000	175.6480	0.7844	0.9163	0.9181
21	303.7690	2.9523	2.0000	226.0140	0.6868	0.6000	0.6363
25	225.2410	2.9486	2.0000	237.9970	0.8119	0.7980	0.8246
27	270.9540	2.9540	3.0000	114.5590	0.4663	0.4806	0.4868
28	360.7500	2.9938	2.0000	159.4810	0.6686	0.7493	0.7844
29	344.3980	2.9933	2.0000	233.2870	0.8661	0.5725	0.6026
3	394.8890	3.1292	3.0000	213.5830	0.2278	0.3097	0.2492
4	375.9440	2.9013	3.0000	206.1430	-0.3290	-0.2851	-0.1862
23	230.4260	2.8838	3.0000	109.1630	0.5023	0.4152	0.4411
24	237.1110	2.8809	2.0000	114.7140	0.7578	0.9378	0.9351
5	304.0900	2.9121	2.0000	333.6530	0.1772	0.1495	0.1355
*Test set*							
7	353.7690	3.0239	2.0000	481.0830	-0.1833	-0.1260	-0.1076
22	228.3700	2.9538	2.0000	313.1860	0.5697	0.5729	0.6014
26	317.0560	2.9527	2.0000	102.8700	1.0589	0.9375	0.9287
2	293.9720	3.0101	2.0000	300.9610	0.7134	0.5724	0.5947
6	400.0460	2.9753	2.0000	309.6650	0.3752	0.1107	0.0919
1	392.1160	3.0356	3.0000	190.2440	0.1772	0.1119	0.0615
11	325.4910	3.0576	2.0000	314.3700	0.9423	0.5740	0.5936
18	298.7220	2.9542	3.0000	88.4821	0.6219	0.4743	0.4797

**Table 8:** Correlation statistics of the model descriptors

Descriptor	ATS7s	SpMax5_Bhv	nHBint6	TDB9m	VIF	Mean effect
ATS7s	1.0000	0.5761	− 0.1319	0.5899	1.7983	− 0.1725
SpMax5_Bhv	0.5761	1.0000	− 0.1931	0.6454	1.9724	1.5138
nHBint6	− 0.1319	− 0.1931	1.0000	− 0.5552	1.6354	− 0.2225
TDB9m	0.5899	0.6454	− 0.5552	1.0000	3.0710	− 0.1187

The Pearson correlation coefficient between pairs of descriptors is less than 0.7 which indicates the independence of descriptors used to build the model. The measure of multicollinearity between the descriptors was computed as variance inflation factor (VIF) as in Eq. 12, where *R*^2^ is the Pearson correlation coefficient for the descriptors.

12 $${\text{VIF}} = {\raise0.7ex\hbox{$1$} \!\mathord{\left/ {\vphantom {1 {\left( {1 - R^{2} } \right)}}}\right.\kern-\nulldelimiterspace} \!\lower0.7ex\hbox{${\left( {1 - R^{2} } \right)}$}}$$

**Fig. 3:**
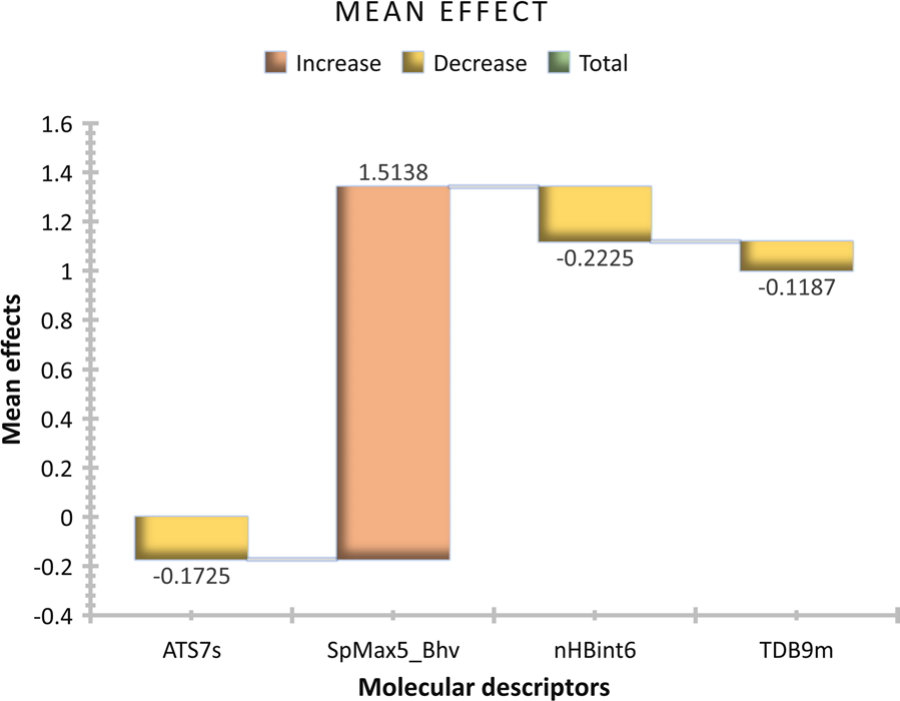
Mean effect plot of the model descriptors

The VIF scores of the four subset descriptors fall within the threshold limit (VIF < 10) suggesting void multicollinearity which implies that each descriptor is orthogonal to one another [41]. The relative contribution of each descriptor toward increase or decrease in the NA inhibitory activity is measured based on their mean effect scores (ME) defined as

13 $${\text{ME}} = \frac{{\beta_{i} \mathop \sum \nolimits_{i}^{n} D_{i} }}{{\mathop \sum \nolimits_{i}^{n} \left( {\beta_{i} \mathop \sum \nolimits_{i}^{n} D_{i} } \right)}}$$

where *βi* represents the coefficient of the descriptor *i*, *Di* represents each descriptor score for a compound, and n represents the number of training set compounds [42]. It was observed that the SpMax5_Bhv is the major contributor to the increase in the NA inhibitory activity with the positive mean effect scores of + 1.538, while the nHBint6, ATS7s, and TDB9m have a negative mean effect of − 0.2225, − 0.1725, and − 0.1187, respectively (Fig. 3). This implies that the increase in the information described by the SpMax5_Bhv descriptor will positively influence the NA activity of the compounds with the decrease in the properties of the remaining descriptors in the model.

**Fig. 4:**
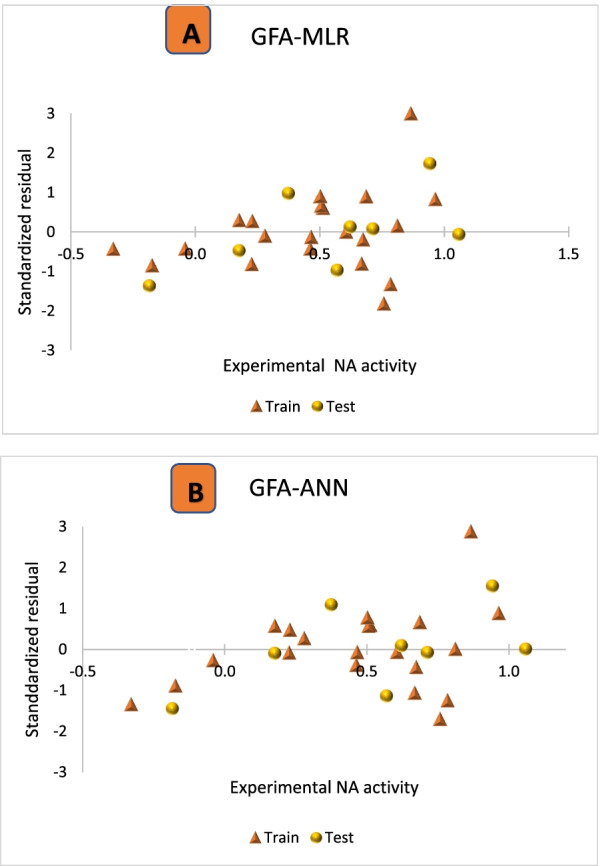
Plot of standardized residuals versus experimental NA activity, **A** GFA-MLR model, **B** GFA-ANN model

The error predictions consist of three important components which include random error (variance), systematic error (bias), and measurement error (noise), but models are more affected by systematic errors [43]. Therefore, a model with high systematic error should be rebuilt to reduce the high level of bias. This is because bias redirects the data into an artificial course that could lead to the wrong interpretation [25]. The ability of the GFA-MLR and GFA-ANN models in predicting the reported NA inhibitory activity of the compounds without any computational errors was assessed using the standardized residual versus NA inhibitory activity plots as shown in Fig. 4A, B. Since all the residual values fall within the definite threshold of ± 2.0, it implies that the model is free of systematic error and can give a good prediction.

#### Model applicability domain of the 2D-QSAR model

**Fig. 5:**
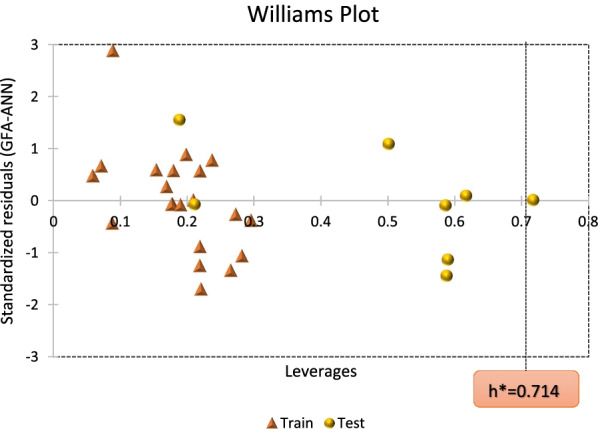
Scatter plot of the standardized residuals of the GFA-ANN model against leverage scores (Williams plot)

The applicability domain of the 2D-QSAR model is the chemical space where the model can make a reliable prediction based on the four selected model descriptors stated earlier. In this study, the leverage approach was applied to examine the chemical space of the GFA-ANN model. The standardized residuals computed were plotted against the leverage values for all compounds (William's plot) to identify the response and structural outliers as presented in Fig. 5. Interestingly, most of the compounds in the dataset were observed to be confined within the standardized residual threshold limit of ± 3.0 and leverage (h*) of 0.714, respectively, except for molecule 26 with a higher leverage score of 0.714 which is inferring that the compound is a structural outlier.

### Statistical validation results for the 3D-QSAR models

**Fig. 6:**
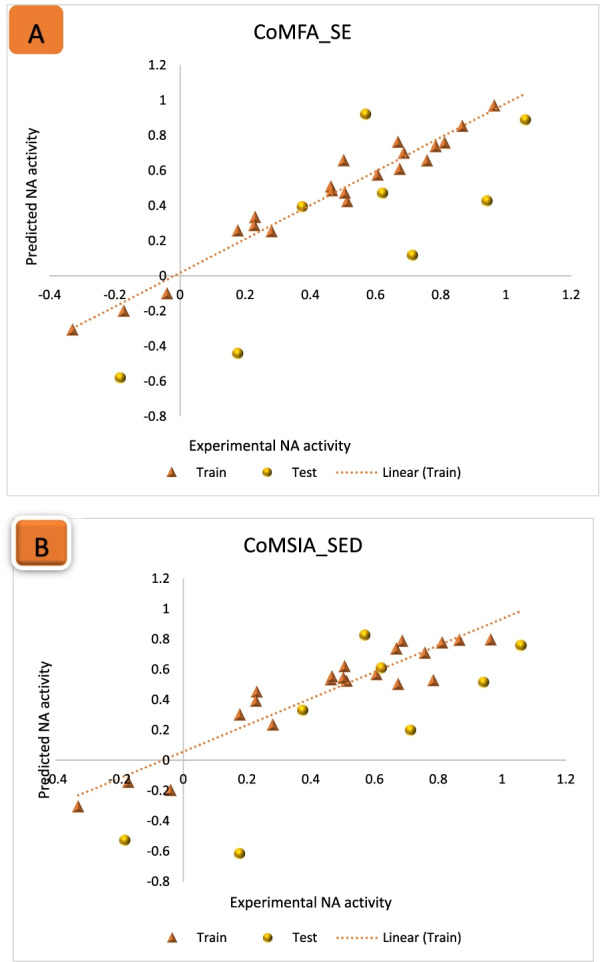
Scatter plot of predicted against experimental NA inhibitory activity: **A** CoMFA_SE model, **B** CoMSIA_SED model

The CoMFA model was built with both electrostatic and steric field contributions as the independent variables of the training set and was further exposed to cross-validation PLS regression analysis and the statistical validation analysis. The statistical validation results of the CoMFA_ES model are *Q*^2^ (0.643 with 4 latent components), non-cross-validated *R*^2^ value (0.962), and SEE (0.0779). However, the statistical validation results from the various probable CoMFA models are summarized in Table 9. For the CoMSIA studies, the statistical validation results of all 30 probable models with different field combinations based on the five field descriptors such as steric (S), electrostatics (E), hydrophobic (H), hydrogen bond donor (D), and hydrogen bond acceptors (A) are shown in Table 10. Furthermore, the CoMSIA-SED model revealed the highest *Q*^2^ score of 0.702 with three components, an *R*^2^ value of 0.877, and a relatively low SEE value of 0.1318. However, the best models among the possible CoMSIA models with robust statistical validation results are summarized in Table 11. In addition, the validations metrics of all possible CoMFA and CoMSIA models were found within the benchmark scores for an acceptable QSAR model that was proposed by Alexander Golbraikh and Alexander Tropsha (*Q*^2^ > 0.5 and *R*^2^ > 0.6). This implies that the validation metrics of the models generated are statistically reliable which indicates their predictive potential and robustness [43]. The graphs of predicted against experimental NA inhibitory activity for the training and test set compounds of the models revealed a satisfactory linear correlation, as presented in Fig. 6 A, B, respectively.

**Table 9:** Statistical validation results of probable CoMFA models

Descriptors	*Q*^2^	*R*^2^	SEE	*N*
Steric (S)	0.315	0.822	0.1584	3
Electrostatic (E)	0.640	0.834	0.1528	3
S + E	0.643	0.962	0.0779	5

*Q*^2^: leave-one-out cross-validated correlation coefficient; *R*^2^: non-cross-validated correlation coefficient; SEE: standard error of estimation; *N*: number of optimum components;

**Table 10:** Statistical validation results of all possible CoMSIA models

S. No.	Descriptors	Q^2^	R^2^	SEE	N
1	Steric (S)	0.639	0.963	0.0771	5
2	Electrostatic (E)	0.662	0.901	0.1216	4
3	Hydrophobic (H)	0.651	0.967	0.0726	5
4	H-Bond Donor(D)	0.659	0.900	0.1223	4
5	H-Bond Acceptor(A)	0.600	0.943	0.0956	5
6	S + E	0.655	0.901	0.1216	4
7	S + H	0.646	0.968	0.0720	5
8	S + D	0.662	0.904	0.1199	4
9	S + A	0.597	0.943	0.0952	5
10	E + H	0.648	0.946	0.0931	5
11	﻿E + D	﻿0.701	﻿0.873	﻿0.1338	﻿3
12	E + A	0.619	0.944	0.0944	5
13	H + D	0.660	0.906	0.1185	4
14	H + A	0.602	0.950	0.0895	5
15	S + E + H	0.647	0.973	0.0682	6
16	﻿S + E + D	0.702	﻿0.877	﻿0.1318	3
17	S + E + A	0.627	0.943	0.0952	5
18	S + H + A	0.600	0.950	0.0894	5
19	S + H + D	0.661	0.909	0.1167	4
20	S + A + D	0.610	0.900	0.1222	4
21	E + A + D	0.662	0.865	0.1378	3
22	E + A + H	0.629	0.951	0.0886	5
23	A + H + D	0.608	0.901	0.1217	4
24	﻿E + D + H	﻿0.699	﻿0.879	﻿0.1306	﻿3
25	S + E + H + D	﻿0.700	﻿0.882	0.1288	﻿3
26	S + E + H + A	0.636	0.950	0.0890	5
27	S + H + D + A	0.661	0.871	0.1350	3
28	E + H + D + A	0.661	0.871	0.1353	3
29	S + E + D + A	0.665	0.870	0.1353	3
30	S + E + H + D + A	0.664	0.875	0.1327	3

**Table 11:** Statistical validation results of the best 3D-QSAR models

3D-QSAR models	*Q*^2^	*R*^2^	SEE	*N*	*R*^2^_test_
CoMFA_E + S	0.643	0.962	0.0779	5	0.6318
CoMSIA_E + D	0.701	0.873	0.1338	3	0.6154
CoMSIA_S + E + D	0.702	0.877	0.1318	3	0.6535
CoMSIA_E + D + H	0.699	0.879	0.1306	3	0.6070
CoMSIA_S + E + D + H	0.700	0.882	0.1288	3	0.6610

#### Contour map analysis of the CoMFA and CoMSIA models

**Fig. 7:**
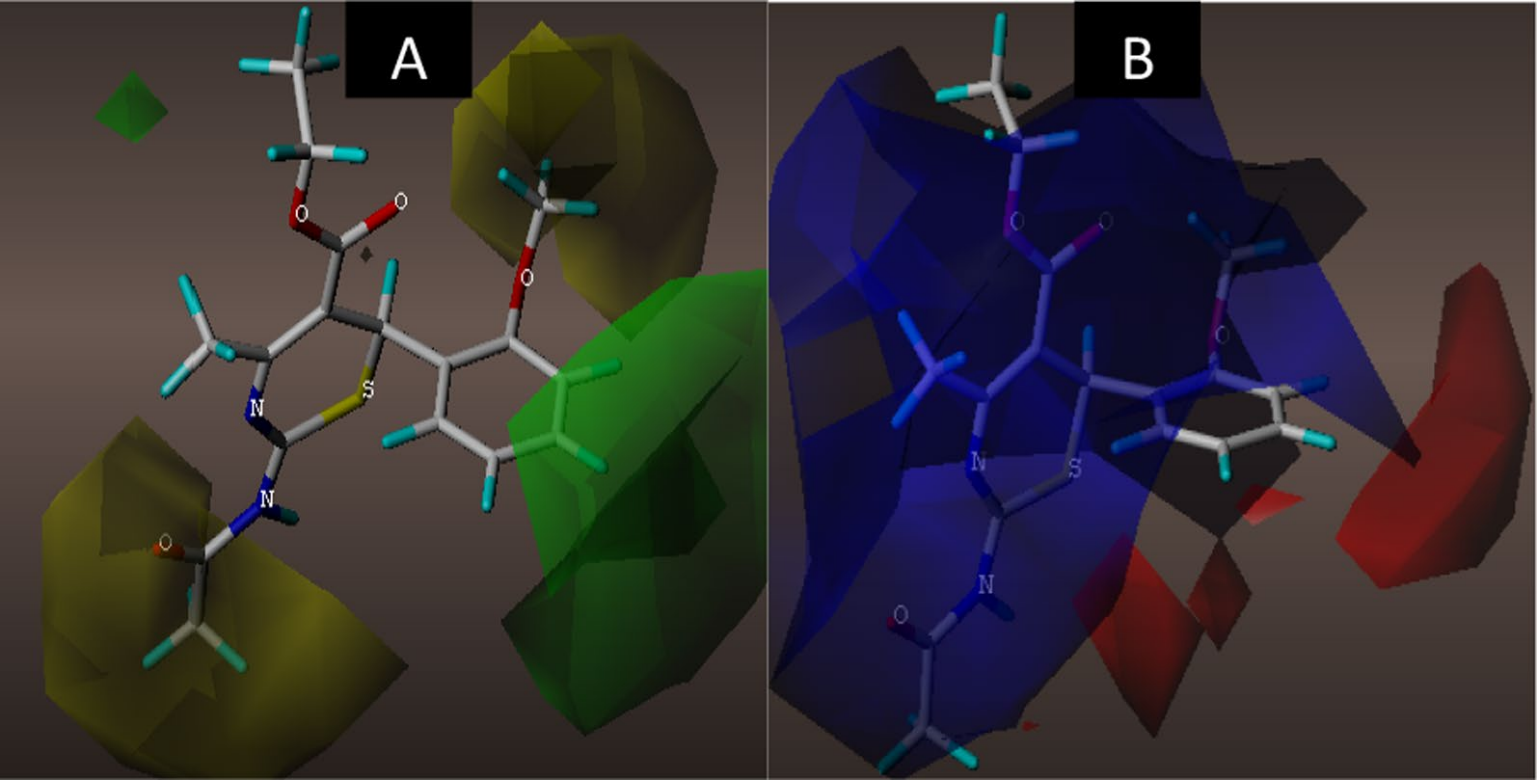
3D fields of the CoMFA model for the most active compound 4. **A** Green areas depict desirable steric bulk, while yellow areas disfavor steric bulk, **B** electrostatic contour map where blue regions favor positive charge and red regions favor negative charge

The utmost advantage of applying CoMFA and CoMSIA approaches is to be able to visualize the field effect of the compound structure on the specific target property in terms of contour maps. These contour maps explicitly identify important regions that are under the influence of some conformational field energies where any changes may significantly affect the target property. Compound 4 as the most potent compound was chosen as a template to examine the most prominent field contributions for the studied dataset. The steric and electrostatic contour maps of the CoMFA_ES model for compound 4 are shown in Fig. 7A, B, respectively. The green and yellow contour maps represent the steric interactions, while the red and blue contours signify electrostatic interactions. The CoMFA contour maps of steric and electrostatic interactions give valuable information on the regions around the molecule that can decrease or increase the NA inhibitory activities. For steric contour maps, the green contour depicts that the desirable addition of bulky groups in the regions would increase the activity, while the yellow contours portrayed that the steric or bulky groups are undesirable in the region for increasing activity [44]. The green contour was predominantly distributed near positions (3, 4, and 5) of the benzene ring, proposing that further addition of bulky groups in these regions would enhance the activity. Meanwhile, the yellow contours near the 2-MeO and the acetamido groups of the same compound suggest that further attachment of bulky fragments in the region would decrease the activity of the compound. In the electrostatic field contour maps, the red regions depict regions where electron-withdrawing groups enhance the activity, while the blue regions depict regions where electron-donating groups increase the activity. The red contour near the meta and para positions of the benzene ring suggests that attaching electronegative groups at the positions may increase the NA inhibitory activities of the compounds.

**Fig. 8:**
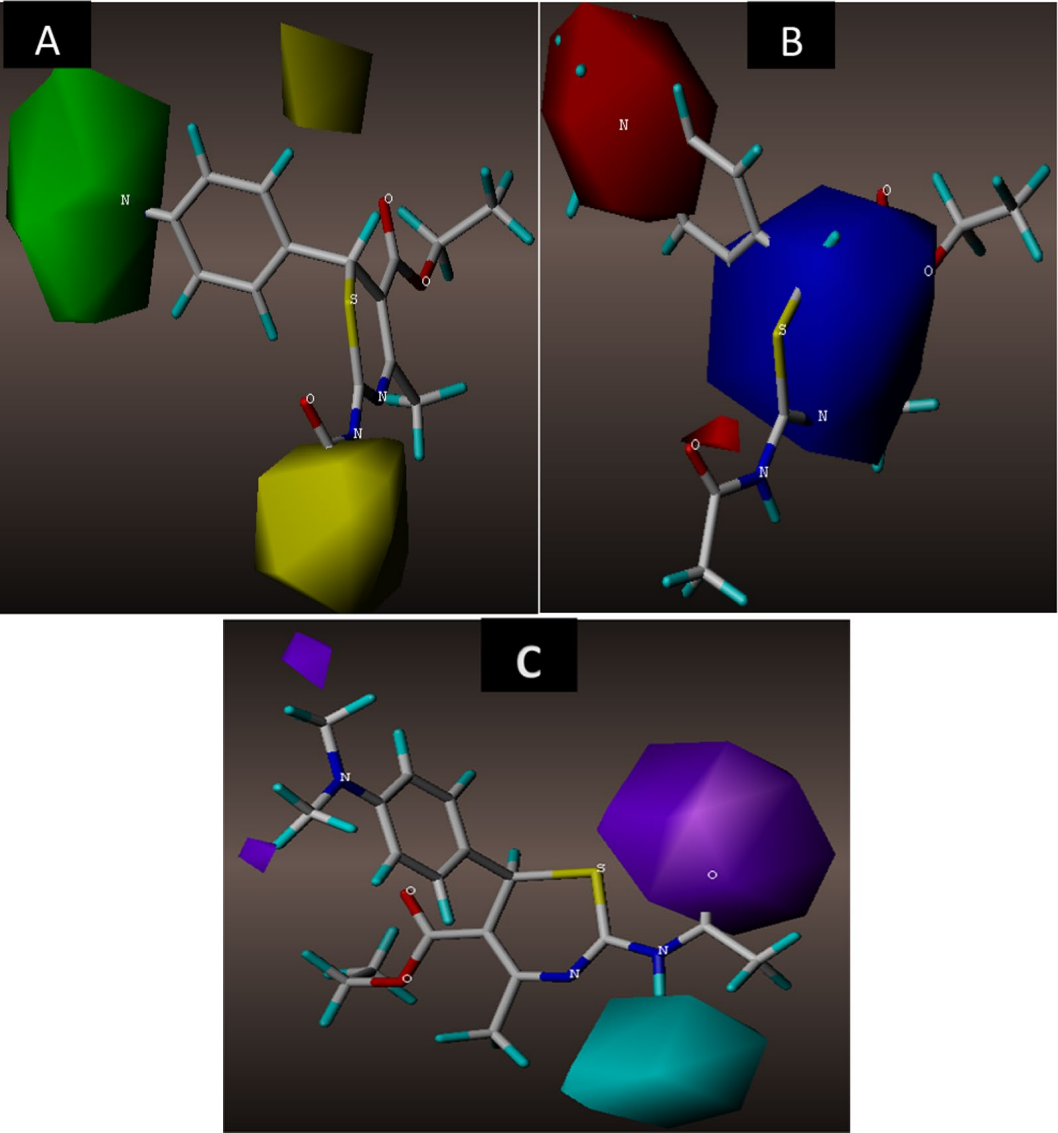
3D fields contribution of the CoMSIA_EAD model for the most active compound 4. **A** Magenta contours represent regions for desirable hydrogen bond acceptors, while red areas represent undesirable acceptors, **B** electrostatic contour map where blue regions favor positive charge and red regions favors negative charge, **C** cyan contours represent areas for desirable hydrogen bond donors, while purple areas represent undesirable donors

The 3D contour maps for the best CoMSIA_SED model are shown in Fig. 8A–C, where the electrostatic contour map in the model is more or less similar to that of the CoMFA model. As such, the discussion will make emphasis on the hydrogen bond fields. The HBD contour map is presented in Fig. 7A, where the cyan contours depict the HBD favorable regions and the purple contour reveals unfavorable HBD regions for the HBD contour map. The purple contour was observed near the carbonyl oxygen (C=O) of the 2-acetamido group, while the cyan contour was embedded near the –HN group of the acetamido group of the same compound.

**Fig. 9:**
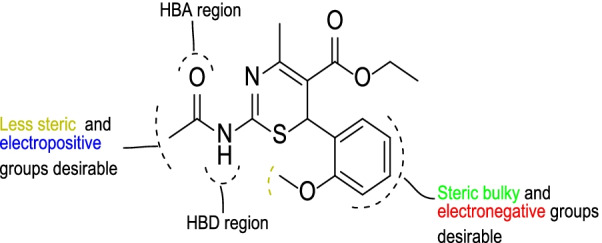
General description of the 3D QSAR analysis

Based on the CoMFA and CoMSIA contour maps analysis, it was observed that the 2-acetamido group, 5-carboxylate group, and the substituents around the benzene ring of the compounds are significant for the NA inhibitory activity as summarized in Fig. 9.

### Molecular docking studies

**Fig. 10:**
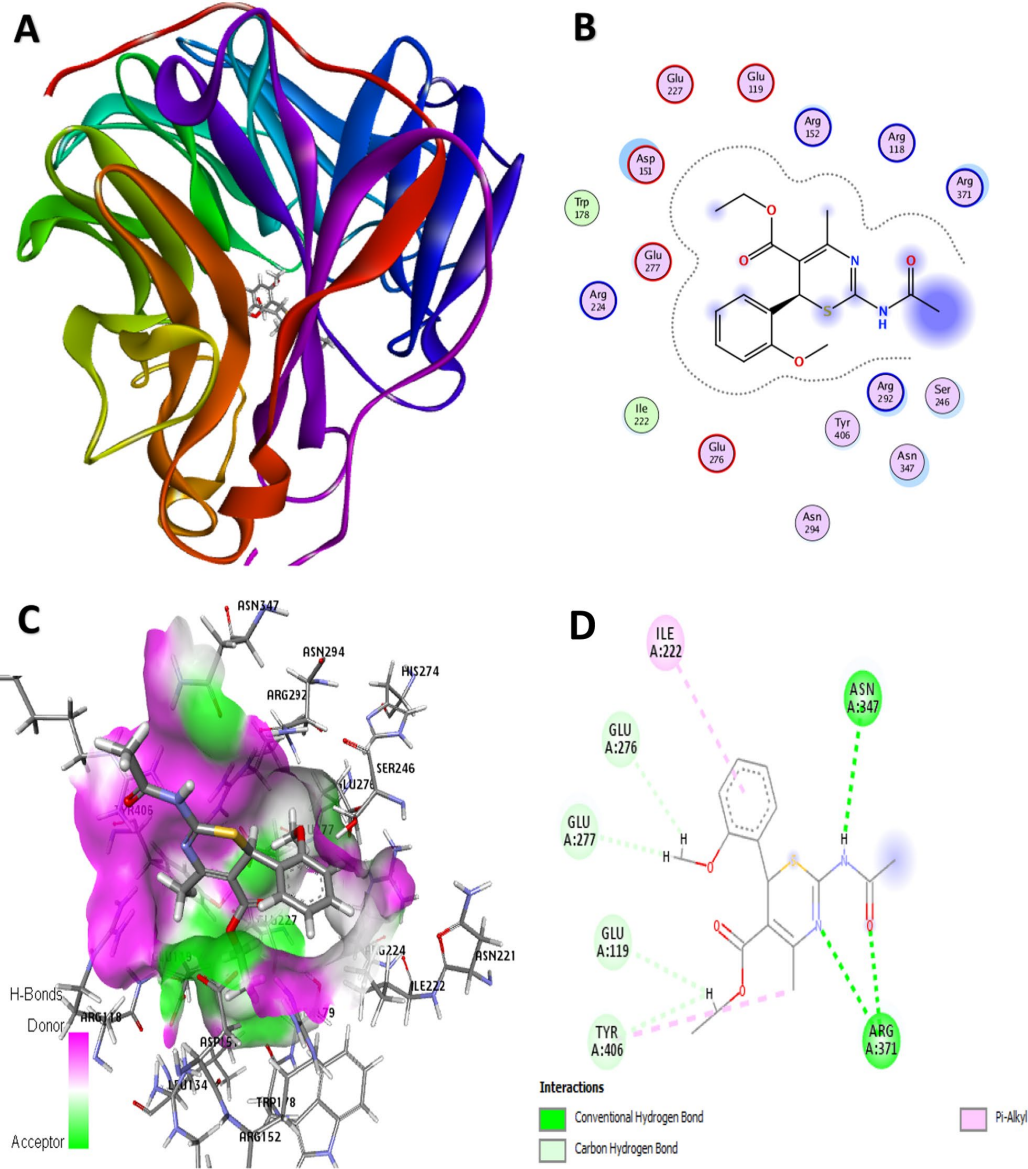
3D docking view of compound 4 with the H1N1 neuraminidase receptor (PDB: 3TI6). **A** The best pose of compound 4, **B** residual interaction of compound 4-complex, **C** 3D hydrogen bond surfaces around the ligand, **D** 2D residual interaction of 4-complex

**Fig. 11:**
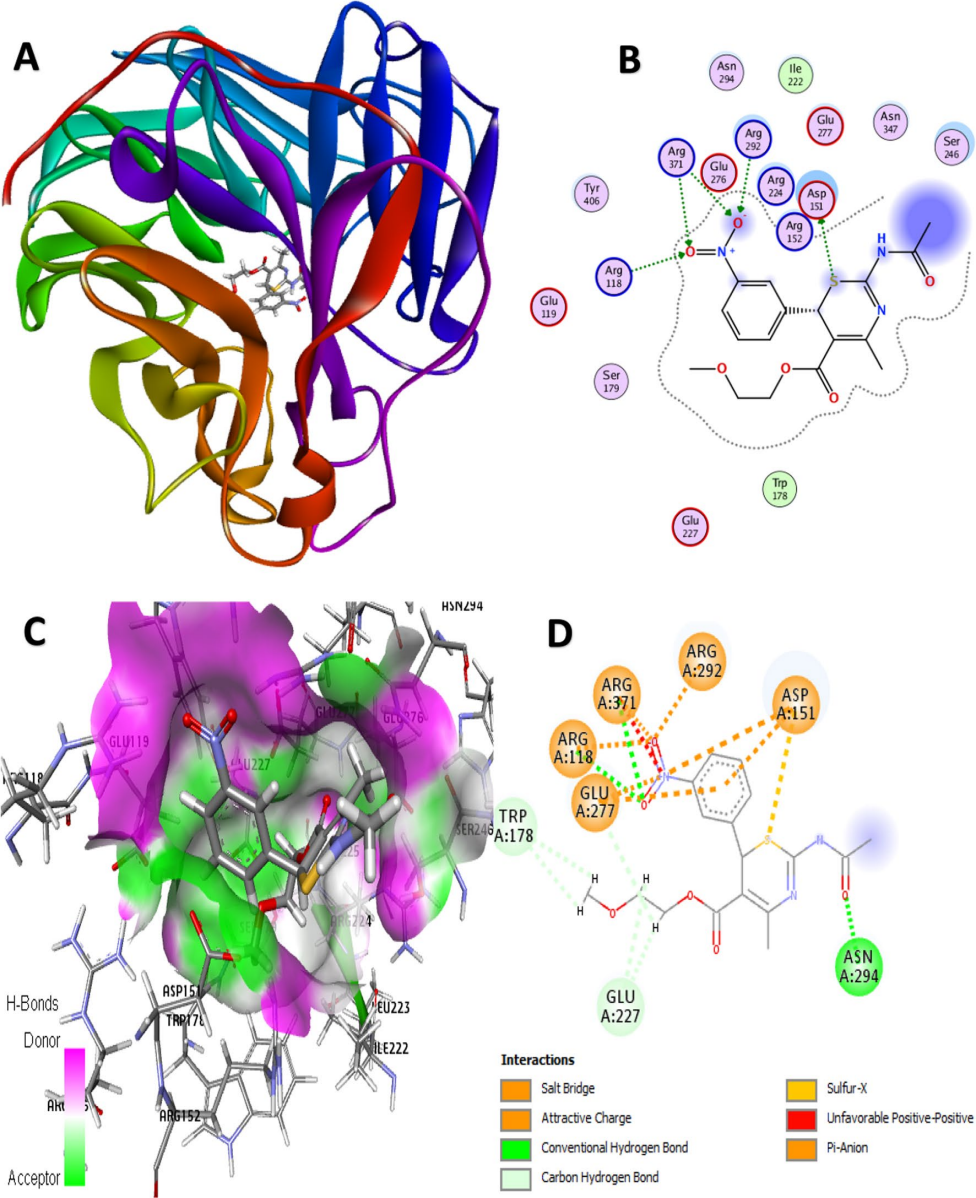
3D docking view of compound 15 with the H1N1 neuraminidase receptor (PDB: 3TI6). **A** The best pose of compound 15, **B** residual interaction of compound 15-complex, **C** 3D hydrogen bond surfaces around the ligand, **D** 2D residual interaction of compound 15-complex

Molecular docking simulation is an important molecular modeling strategy in the computer-assisted design of new compounds (structure-based drug design) which provides information about the residual interaction types of target ligands with the active site of a protein as a receptor [28]. Before the docking started, the co-crystallized oseltamivir was extracted from the H1N1 neuraminidase protein (PDB: 3TI6) and then docked into the binding pocket to confirm the reliability of the docking algorithm used by the MOE program as well as to note the amino acid residues surrounding the ligand. The results revealed the docking scores of the best poses ranging from − 7.2004 to − 5.8457 kcal/mol as shown in Table 12. From the docking score results, four lead compounds (4, 7, 14, and 15) with a relatively high inhibitory rate (> 50%) and docking score (> − 6.3 kcal/mol) were identified as the possible lead candidates for future exploration of improved anti-influenza agents. Compound 4 as the most potent molecule with an activity of 68.9% had a good docking score of − 6.3290 kcal/mol, and the residual profile of the complex is presented in Fig. 10. For the conventional H-bond interaction analysis, the residue of ARG371 behaves as H-bond donors to the carbonyl oxygen (C=O) and nitrogen of the thiazine core of the molecule 4 as the H-bond acceptors to form 3 hydrogen bond interactions, while the hydrogen (–HN) of the acetamido group behaves as H-bond donor to the oxygen atom of ASN 347 residue at a bond distance of 2.9575 Å as summarized in Table 13. The hydrogen atoms of the methoxy (2-MeO) and ethyl groups of the same compound behave as H-bond donors to the oxygen atoms of the ASN347, GLU277, GLU276, and GLU119 as the acceptors to form C–H bond interactions at different bond distances. For the hydrophobic interactions, the *π*-orbital of the Tyr 406 residue interacts with the methyl (alkyl) group of the same compound to form a *π*-alkyl hydrophobic interaction type, while the other hydrophobic interaction formed was due to the *π*-orbital interaction from the compound with an alkyl group of ILE 222 residue at different distances, respectively (Fig. 11).

**Table 12:** Molecular docking scores of the 1,3-thiazine derivatives

S. No.	Score	rmsd_refine	E_conf	E_place	E_score1	E_refine	E_score2
1	− 6.4901	1.3386	− 122.1681	− 48.5505	− 9.4307	− 25.8249	− 6.4901
2	− 6.5735	1.8070	− 120.4932	− 70.4411	− 9.9137	− 33.2956	− 6.5735
3	− 6.3551	1.5179	− 150.6771	− 50.2391	− 10.2081	− 36.4173	− 6.3551
4 ﻿	− 6.3290	1.6909	﻿− 152.5328	﻿− 77.3417	﻿− 9.7922	﻿− 33.5268	﻿− 33.5268
5	− 5.8457	0.8892	− 149.0868	− 38.0634	− 9.2550	− 21.9753	− 5.8457
6	− 6.5115	1.3100	− 134.0408	− 71.9441	− 10.3233	− 20.1000	− 6.5115
7	﻿− 6.6258	﻿1.5226	﻿− 121.8536	− 73.6325	﻿− 9.7901	﻿− 28.5852	− 6.6258
8	− 6.3074	2.6590	− 141.4917	− 32.7300	− 10.0024	− 31.0956	− 6.3074
9	− 6.5875	1.1095	− 164.7347	− 70.6521	− 9.4724	− 25.4637	− 6.5875
10	− 6.1763	1.9554	− 139.1840	− 50.2221	− 9.3584	− 28.1367	− 6.1763
11	− 6.2310	2.9689	− 154.7956	− 44.6518	− 9.9946	− 36.2267	− 6.2310
12	− 6.3392	1.7148	− 152.7194	− 51.0108	− 10.1836	− 30.4122	− 6.3392
13	− 7.2004	1.3127	− 163.7089	− 83.6582	− 9.7403	− 34.5889	− 7.2004
14	﻿− 6.8369	﻿1.4159	﻿− 149.8267	﻿− 62.5150	﻿− 10.2103	﻿− 31.9916	﻿− 6.8369
15	﻿− 6.8435	﻿1.0493	﻿− 108.2935	﻿− 53.3988	﻿− 10.0980	﻿− 33.9323	﻿− 6.8435
16	− 6.2685	0.9614	− 95.1933	− 64.1636	− 9.9163	− 22.2508	− 6.2685
17	− 6.5204	0.8028	− 94.8014	− 84.4307	− 10.1112	− 29.5498	− 6.5204
18	− 6.5349	1.2536	− 130.5752	− 70.3961	− 10.2722	− 26.5739	− 6.5349
19	− 6.2513	1.2438	− 127.6415	− 63.2090	− 10.4032	− 31.3327	− 6.2513
20	− 5.9769	2.0611	− 111.9184	− 55.1487	− 10.4111	− 24.8607	− 5.9769
21	− 6.5886	1.2004	− 106.8793	− 83.1832	− 12.7530	− 37.4622	− 6.5886
22	− 6.3499	1.0607	− 126.4274	− 66.8849	− 9.6530	− 30.2890	− 6.3499
23	− 6.1877	1.4714	− 132.0677	− 60.9961	− 11.8590	− 26.3517	− 6.1877
24	− 6.0548	1.3597	− 112.9793	− 60.1168	− 9.4625	− 28.6708	− 6.0548
25	− 6.2808	1.4735	− 127.5015	− 66.0587	− 9.6263	− 30.4590	− 6.2808
26	− 6.1317	1.5469	− 131.9421	− 60.5972	− 9.9004	− 25.7525	− 6.1317
27	− 6.2819	2.1607	− 138.3339	− 79.3915	− 11.5250	− 27.2055	− 6.2819
28	− 6.6802	1.2019	− 120.7970	− 65.8939	− 10.1814	− 39.3712	− 6.6802
29	− 6.4982	1.4052	− 73.6767	− 63.0311	− 10.3986	− 28.9355	− 6.4982
Oseltamivir	− 9.2388	1.3910	− 152.6088	− 74.5023	− 17.5359	− 65.9785	− 9.2388

Score: the final docking score, rmsd_refine: the root-mean-square deviation between the pose before and after refinement, E_conf: the energy of the conformer. E_refine: core from the refinement stage, calculated to be the sum of the van der Waals electrostatics and solvation energies, under the generalized Born solvation model (GB/VI), E_score1: score from rescoring stages 1, E_place: score from the placement stage, E_score2: score from rescoring stages 2

**Table 13:** Binding interaction of the H1N1 neuraminidase receptor with compound 4

Bond (Å)	Interaction type	From	Chemistry	To	Chemistry
2.9723	Hydrogen Bond	A: ARG371	H-Donor	4	H-Acceptor
2.7175	Hydrogen Bond	A: ARG371	H-Donor	4	H-Acceptor
2.6552	Hydrogen Bond	A: ARG371	H-Donor	4	H-Acceptor
2.9575	Hydrogen Bond	4	H-Donor	A: ASN347	H-Acceptor
2.5011	Carbon Hydrogen Bond	4	H-Donor	A: ASN347	H-Acceptor
2.4892	Carbon Hydrogen Bond	4	H-Donor	A: GLU277	H-Acceptor
2.6665	Carbon Hydrogen Bond	4	H-Donor	4	H-Acceptor
2.5644	Carbon Hydrogen Bond	4	H-Donor	A: GLU276	H-Acceptor
2.6332	Carbon Hydrogen Bond	4	H-Donor	A: GLU119	H-Acceptor
5.1658	Hydrophobic(π-alkyl)	A: TYR406	*π*-orbital	4	Alkyl
5.3130	Hydrophobic(π-alkyl)	4	*π*-orbital	A: ILE222	Alkyl

**Fig. 12:**
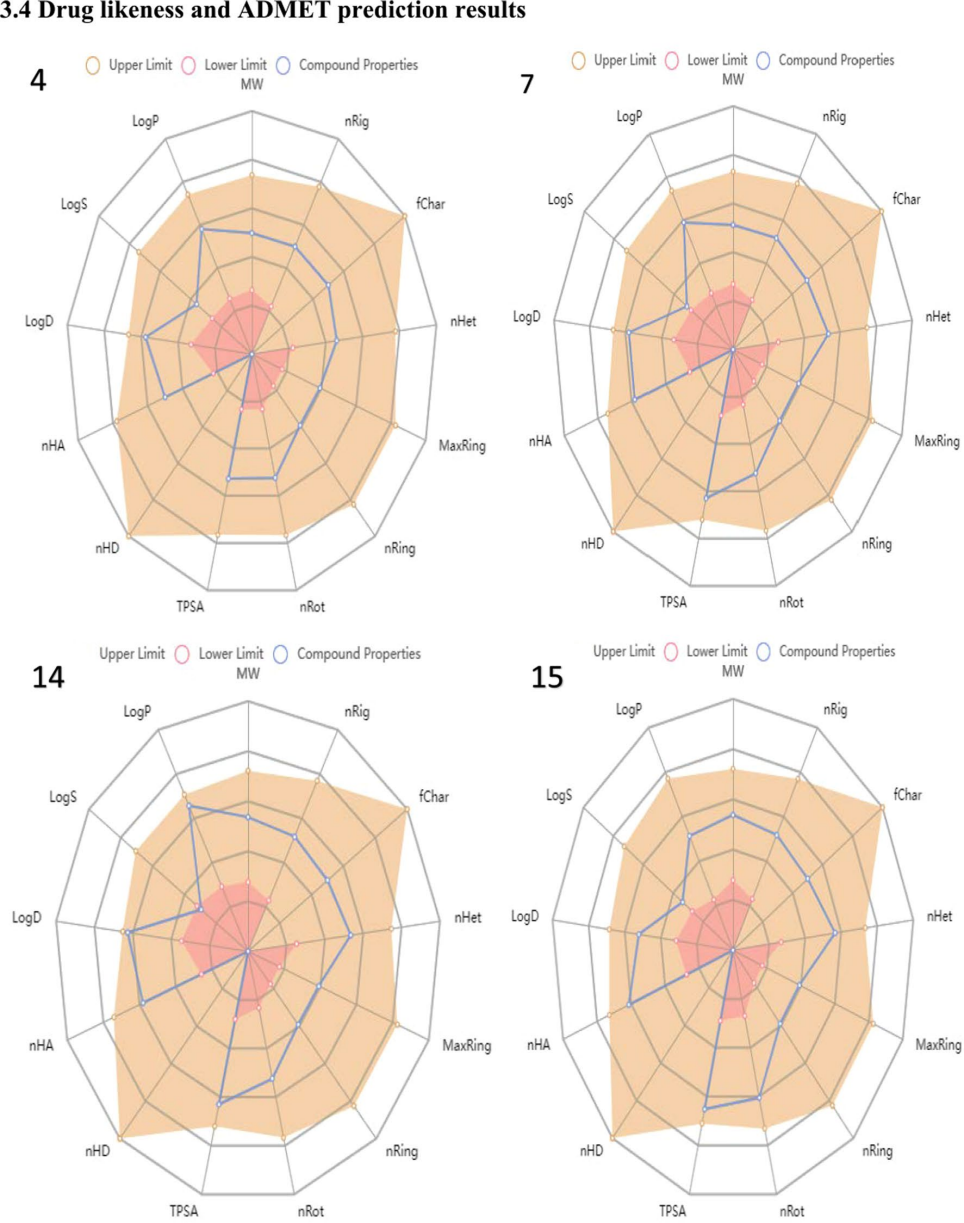
Physicochemical radar chart of the lead compounds in the dataset

The major residual interactions in 15-complex (Fig. 12) with the highest binding score of − 6.8435 kcal/mol include four conventional H-bonds, five C–H bonds, five electrostatic interactions, and two salt bridges with different amino acid residues in the NA active site of the targeted receptor, which are summarized in Table 14. For the conventional H-bond interaction, the active residues of ARG118, ASN294, and ARG371 behave as H-bond donors to the oxygens from the nitro (NO_2_) and carbonyl (C=O) group of the compound 15. Also, the hydrogen atoms of the –(CH_2_)_2_OCH_3_ moiety of the same compound behave as H-bond donors to the oxygen atoms of the GLU227 and TRP178 as the acceptors to form the carbon–H bond interactions at different bond distances. The amino acid residues of ASP151, GLU277, and ARG118 interact with the π-orbital of the same compound to form π-anion electrostatic interactions, while the partial positive charge of the nitro group (3-NO_2_) interacts with the negative charges of ASP151 and GLU277 residues to form attractive charge interactions (electrostatic). In addition, the residues of ARG292 and ARG371 also formed two salt bridges (a combination of electrostatic and hydrogen bond interactions) with oxygen from the nitro group of the compound at different bond distances.

**Table 14:** Binding residual interaction of the H1N1 neuraminidase receptor with compound 15

Bond (Å)	Interaction type	From	Chemistry	To	Chemistry
3.0716	Carbon hydrogen bond	15	H-Donor	A: GLU227	H-Acceptor
2.7803	Carbon hydrogen bond	15	H-Donor	A: GLU277	H-Acceptor
2.3958	Carbon hydrogen bond	15	H-Donor	A: GLU227	H-Acceptor
2.7646	Carbon hydrogen bond	15	H-Donor	A: TRP178	H-Acceptor
2.5774	Carbon hydrogen bond	15	H-Donor	A: TRP178	H-Acceptor
1.9756	Hydrogen bond	A: ARG118	H-Donor	15	H-Acceptor
2.0478	Hydrogen bond	A: ARG118	H-Donor	15	H-Acceptor
2.5643	Hydrogen bond	A: ASN294	H-Donor	15	H-Acceptor
1.9466	Hydrogen bond	A: ARG371	H-Donor	15	H-Acceptor
3.1851	Other (Sulfur-X)	15	Sulfur	A: ASP151	Sulfur
2.0022	Electrostatic; H-bond	A: ARG292	Positive; H-Donor	15	Negative; H-Acceptor
1.9585	Electrostatic; H-bond	A: ARG371	Positive; H-Donor	15	Negative; H-Acceptor
4.6885	Electrostatic	A: ARG118	Positive	15	Negative
4.6762	Electrostatic	15	Positive	A: ASP151	Negative
4.4499	Electrostatic	15	Positive	A: GLU277	Negative
3.0763	Electrostatic	A: ASP151	Negative	15	*π*-orbital
3.9660	Electrostatic	A: GLU277	Negative	15	*π*-orbital

### Drug-likeness assessment and ADMET predictions

In evaluating the drug-likeness of the compounds, their physicochemical properties are usually related to some filter variants. Therefore, relevant physicochemical parameters **(**Fig. 12) are generated from the ADMETlab 2.0 Web server. The physicochemical properties for the four lead compounds (4, 7, 14, and 15) are within the upper limit (brown) and lower limit (red) as presented in the radar charts accordingly (Fig. 12). The four lead compounds which have passed the Lipinski rule of five (Table 10) were further assessed with other drug-likeness filter rules such as the Ghose filter rule, Veber’s rule, Egan’rule, and Muegge’s rule using the SwissADME Web server as shown in Table 15. Lipinski's criteria for drug-likeness include molecular weight (MW ≤ 500 g/mol), n-octanol/water distribution coefficient (Log *P* ≤ 5), number of hydrogen bond acceptors (nHA ≤ 10), and number of hydrogen bond donors (nHD ≤ 5) [45, 46]. From Lipinski's table of the lead compounds in the dataset, the Log P scores of the compounds are relatively high close to 3 log mol/L which is the optimal limit (0 < log *P* < 3). This implies that the compounds have low aqueous solubility and good oral bioavailability [47, 48]. The Log *P* also gives information on the cellular membrane permeability and hydrophobic binding to macromolecules such as the target receptors, plasma proteins, metabolizing enzymes, or transporters [49]. Oseltamivir as the standard neuraminidase drug has lower log *P* scores of − 1.317 which tend to experience difficulty in penetrating the lipid bilayer of the cell membrane. The lead compounds were appraised by other drug-likeness rules such as Ghose, Veber, Egan, and Muegge rules, and the result depicted that they all satisfied most of the rules except for compounds 7and 15 which have violated Egan rule as shown in Table 16.

**Table 15:** Lipinski’s rule of the lead compounds in the dataset

S. No.	MW (g/mol)	Log *P* (log mol/L)	nHA	nHD	TPSA	nLV
4	348.11	2.003	6	0	77.320	0
7	363.09	2.067	8	0	111.230	0
14	391.12	2.637	8	0	111.230	0
15	393.10	1.575	9	0	120.46	0
Oseltamivir	330.15	− 1.317	10	9		0
Rule	≤ 500	≤ 5	≤ 10	≤ 5		≤ 1

Key: Molecular weight (MW), n-octanol/water distribution coefficient (Log P), number of hydrogens bond acceptors (nHA), number of hydrogen bond donors (nHD), number of Lipinski violations (nLV)

**Table 16:** Drug-likeness assessment of the lead compounds based on Ghose, Veber, Egan, and Muegge

S. No.	Ghose	Veber	Egan	Muegge
4	Yes	Yes	Yes	Yes
7	Yes	Yes	No	Yes
14	Yes	Yes	Yes	Yes
15	Yes	Yes	No	Yes
Oseltamivir	Yes	Yes	Yes	Yes

The biochemical processes involved from the administration of a drug into the body to its elimination play an important role in lead identification and optimization [50]. A perfect drug candidate must be non-toxic, and when administered should be absorbed into the circulatory system and eradicated without affecting the biological activity [50]. These discrete biochemical processes are closely interrelated, leading to the evaluation of ADMET properties as one of the prime factors in the process of drug discovery [51].

Some of the relevant computed ADMETlab 2.0 parameters generated include human intestinal absorption (HIA), human colon adenocarcinoma cell lines (Caco-2) permeability, Madin–Darby canine kidney cells (MDCK) permeability, plasma glycoprotein (Pgp) inhibitor, plasma glycoprotein (Pgp) substrate, plasma protein binding (PPB), volume distribution (VD), blood–brain barrier (BBB) penetration, human cytochromes (CYP), clearance (CL), half-life (T1/2), AMES toxicity, carcinogenicity (Carc), eye irritation (EI), and respiratory toxicity (RT) as shown in Table 17. The computed value for HIA showed that the lead compounds have the probability of excellent absorption from the intestinal membrane. The Caco-2 cell permeability has been an important index for an eligible drug candidate which is associated with human intestinal absorption [46]. The lead compounds were considered to have proper Caco-2 cell permeability because their values are higher than the optimal score of − 5.15 log cm/s. The MDCK permeability is utilized as an in vitro model for permeability screening, and its apparent coefficient is used to assess the efficiency of chemicals in the body and also to estimate the effect of the blood–brain barrier. The lead compounds were considered to have high passive MDCK permeability with predicted coefficients of greater than 2.0 × 10^−5^ cm/s. The output results of the lead compounds revealed an excellent probability of being Pgp substrates. PPB is one of the most important mechanisms of drug uptake and distribution resulting from the drug–protein bindings in the plasma which strongly affects the pharmacodynamics behavior of the drug [52]. The lead compounds were also predicted to have a high value of PPB (> 90%) depicting a broad therapeutic index. The theoretical concept of the VD parameter is used to relate the administered drug dose with the actual initial concentration in the circulatory system which often describes the in vivo distribution [52]. As such, the lead compounds are predicted to have proper VD values in the range of 0.04–20 L/kg. The BBB permeate output of the lead compounds predicted no BBB penetration may cause any central nervous system side effects. For the metabolism, the predicted outputs revealed the probabilities of being either lead substrates or inhibitors of CYPs of the isoenzymes (1A2, 3A4, 2C9, 2C19, and 2D6) whose range of values is within 0 to 1. The clearance of a drug (CL) is an important pharmacokinetic measure that describes how the drug is excreted from the body. The predicted clearance penetration results of the lead compounds showed that compounds **4, 14, and 15** are predicted to have low clearance levels, while compound 7 tends to have moderate clearance (< 5 mg/min/kg). In terms of toxicity, the AMES mutagenicity, eye irritation, and respiratory toxicity of the lead compounds are mostly predicted as non-toxic which is in agreement with the previous reports.

**Table 17:** ADMET properties of the lead compounds

Category	Properties	Prediction probability values (symbols)			
		4	7	14	15
Absorption	Human intestinal absorption	0.005 (---)	0.004(---)	0.002(---)	0.005(---)
	Caco-2 permeability	− 4.802	− 4.823	− 4.859	− 4.91
	MDCK permeability	1.81 × 10^−5^	1.86 × 10^−4^	1.42 × 10^−4^	1.15 × 10^−4^
	Pgp-inhibitor	0.837 (++)	0.213(--)	0.795(++)	0.363(-)
	Pgp-substrate	0.001 (---)	0.001(---)	0 (---)	0.002(---)
	Protein plasma binding	80.44%	80.94%	93.35%	71.13%
Distribution	Volume distribution	0.857	0.885	1.051	0.872
	BBB penetration	0.983 (+++)	0.883(++)	0.826 (++)	0.832(++)
	CYP1A2 inhibitor	0.546 (+)	0.191 (--)	0.223 (--)	0.066 (---)
	CYP1A2 substrate	0.391 (-)	0.107 (---)	0.088 (---)	0.506 (+)
	CYP2C19 inhibitor	0.407 (-)	0.531(+)	0.522 ( +)	0.21 (--)
	CYP2C19 substrate	0.913 (+++)	0.757(++)	0.812(++)	0.614(+)
Metabolism	CYP2C9 inhibitor	0.165 (--)	0.139(+)	0.615 (+)	0.075(---)
	CYP2C9 substrate	0.21 (--)	0.333(--)	0.117 (--)	0.081(---)
	CYP2D6 inhibitor	0.001 (---)	0.003(---)	0.006 (---)	0.002(---)
	CYP2D6 substrate	0.22 (--)	0.184(--)	0.143 (--)	0.134 (--)
	CYP3A4 inhibitor	0.322 (-)	0.212(-)	0.423 (-)	0.231(--)
	CYP3A4 substrate	0.776 (++)	0.586(+)	0.576(+)	0.564 (+)
Excretion	Clearance level	7.405	4.806	8.948	6.861
	Half-life	0.476	0.271	0.327	0.387
	AMES toxicity	0.036 (---)	0.652 (--)	0.188 (--)	0.650 (+)
Toxicity	Carcinogenicity	0.095 (---)	0.367 (++)	0.704 (++)	0.485 (-)
	Eye irritation	0.015 (---)	0.014 (---)	0.012 (---)	0.012 (---)
	Respiratory toxicity	0.327 (-)	0.567 (---)	0.691 (+)	0.573 (+)

Key: The prediction probability values are classified into six symbols: 0-0.1(---), 0.1–0.3(--), 0.3–0.5(-), 0.5–0.7(+), 0.7–0.9(++), and 0.9–1.0(+++). Generally, ‘+++’ or ‘++’ represents that the compounds are more likely to be toxic or defective, while ‘– –’or ‘–’ represents non-toxic or appropriate

## Conclusion

In conclusion, the study utilized computational modeling concepts such as 2D-QSAR, 3D-QSAR, molecular docking, and ADMET predictions of 29 analogs of 1,3-thiazine derivatives as influenza neuraminidase inhibitors to explore the various leads for exploration of improved compounds. The GFA-MLR and GFA-ANN models with feature selected descriptors, ATS7s, SpMax5_Bhv, nHBint6, and TDB9m, were found to have reliable prediction of the NA inhibitory activities from the 2D-QSAR modeling studies. The 3D-QSAR studies further revealed the correlation of various conformational fields as a function of NA inhibitory activity of the compounds from the previewed contour maps of the CoMFA and CoMSIA models. The statistical validation of the 2D-QSAR and 3D-QSAR was all within the global benchmarks for accepting QSAR models which supports the predictive performance of the models. The drug-likeness and ADMET predictions of the lead compounds revealed non-violation of Lipinski’s rule and good pharmacokinetic profiles, respectively, as essential guidelines for rational drug design. The outcome of this study overlaid a solid foundation for the in silico design and exploration of novel NA inhibitors with improved potency.

## Data Availability

Data sharing is not applicable to this article as no datasets were generated or analyzed during the current study.

## Abbreviations

MLR: Multi-linear regression
QSAR: Quantitative structure–activity relationship
ANN: Artificial neural network
GFA: Genetic function approximation
NA: Neuraminidase
PLS: Partial least squares
2D-QSAR: Two-dimensional quantitative structure–activity relationship
3D-QSAR: Three-dimensional quantitative structure–activity relationship
NA: Neuraminidase

## References

[1] Akhtar Z, Islam MA, Aleem MA, Mah EMS, Ahmmed MK, Ghosh PK (2021). SARS-CoV-2 and influenza virus coinfection among patients with severe acute respiratory infection during the first wave of COVID-19 pandemic in Bangladesh: a hospital-based descriptive study. BMJ Open.

[2] Binns E, Koenraads M, Hristeva L, Flamant A, Baier-Grabner S, Loi M (2022). Influenza and respiratory syncytial virus during the COVID-19 pandemic: time for a new paradigm?. Pediatr Pulmonol.

[3] Lawson A, López-Candales A (2022). COVID-19 and seasonal influenza. Postgrad Med.

[4] Aouissi HA, Ababsa M, Leveau CM, Petrisor A-I, Słomka A, Kechebar MSA, et al. Beyond vaccination: a Cross-Sectional Study of the importance of Behavioral and Native Factors on COVID-19 Infection and Severity. medRxiv. 2022:2022.01.23.22269214 10.3390/healthcare10071341 PMC9323463 35885867

[5] Olsen SJ, Winn AK, Budd AP, Prill MM, Steel J, Midgley CM (2021). Changes in influenza and other respiratory virus activity during the COVID-19 Pandemic: United States, 2020–2021. MMWR Morb Mortal Wkly Rep.

[6] McKimm-Breschkin JL, Hay AJ, Cao B, Cox RJ, Dunning J, Moen AC (2022). COVID-19, Influenza and RSV: Surveillance-informed prevention and treatment: meeting report from an isirv-WHO virtual conference. Antiviral Res.

[7] Dadashi M, Khaleghnejad S, AbediElkhichi P, Goudarzi M, Goudarzi H, Taghavi A (2021). COVID-19 and influenza co-infection: a systematic review and meta-analysis. Front Med.

[8] Biggerstaff M, Kniss K, Jernigan DB, Brammer L, Bresee J, Garg S (2017). Systematic assessment of multiple routine and near real-time indicators to classify the severity of influenza seasons and pandemics in the United States, 2003–2004 Through 2015–2016. Am J Epidemiol.

[9] Sun LH (2018) Last year's flu broke records for deaths and illnesses, new CDC numbers show. The Washington Post

[10] Korsten K, Adriaenssens N, Coenen S, Butler CC, Verheij TJM, Bont LJ (2021). World Health Organization influenza-like illness underestimates the burden of respiratory syncytial virus infection in community-dwelling older adults. J Infect Dis.

[11] Aleebrahim-Dehkordi E, Molavi B, Mokhtari M, Deravi N, Fathi M, Fazel T (2022). T helper type (Th1/Th2) responses to SARS-CoV-2 and influenza A (H1N1) virus: from cytokines produced to immune responses. Transpl Immunol.

[12] Bell R, Imai S, Rafferty A, Little NRG, Winterbauer N, Luo H (2021). Influenza and pneumonia vaccinations among north carolina adults with diabetes. Am J Health Behav.

[13] Aouissi HA, Belhaouchet I (2021). What about rheumatic diseases and COVID-19?. New Microb New Infect.

[14] Hulme KD, Noye EC, Short KR, Labzin LI (2021). Dysregulated inflammation during obesity: driving disease severity in influenza virus and sars-cov-2 infections. Front Immunol.

[15] Abed Y, Bouhy X, L'Huillier AG, Rheaume C, Pizzorno A, Retamal M (2016). The E119D neuraminidase mutation identified in a multidrug-resistant influenza A(H1N1)pdm09 isolate severely alters viral fitness in vitro and in animal models. Antiviral Res.

[16] Avila G, Cruz-Licea V, Rojas-Espinosa K, Bermudez-Alvarez Y, Grostieta E, Romero-Valdovinos M (2020). Influenza A H1N1 Virus 2009 synthetic hemagglutinin and neuraminidase peptides for antibody detection. Arch Med Res.

[17] Adams SE, Lee N, Lugovtsev VY, Kan A, Donnelly RP, Ilyushina NA (2019). Effect of influenza H1N1 neuraminidase V116A and I117V mutations on NA activity and sensitivity to NA inhibitors. Antiviral Res.

[18] Hayden FG, Asher J, Cowling BJ, Hurt AC, Ikematsu H, Kuhlbusch K (2022). Reducing influenza virus transmission: the potential value of antiviral treatment. Clin Infect Dis.

[19] Abdullahi M, Das N, Adeniji SE, Usman AK, Sani AM (2021). In-silico design and ADMET predictions of some new imidazo [1, 2-a] pyridine-3-carboxamides (IPAs) as anti-tubercular agents. J Clin Tuberc Other Mycobact Dis.

[20] Li W, Xia L, Hu A, Liu A, Peng J, Tan W (2013). Design and synthesis of 4-alkyl-2-amino(acetamino)-6-aryl-1,3-thiazine derivatives as influenza neuraminidase inhibitors. Arch Pharm.

[21] Abdullahi M, Shallangwa GA, Uzairu A (2020). In silico QSAR and molecular docking simulation of some novel aryl sulfonamide derivatives as inhibitors of H5N1 influenza A virus subtype. Beni-Suef Univ J Basic Appl Sci.

[22] Abdullahi M, Adeniji SE, Arthur DE, Musa S (2020). Quantitative structure-activity relationship (QSAR) modelling study of some novel carboxamide series as new anti-tubercular agents. Bull Natl Res Centre.

[23] Ahamad S, Islam A, Ahmad F, Dwivedi N, Hassan MI (2019). 2/3D-QSAR, molecular docking and MD simulation studies of FtsZ protein targeting benzimidazoles derivatives. Comput Biol Chem.

[24] Apablaza G, Montoya L, Morales-Verdejo C, Mellado M, Cuellar M, Lagos CF (2017). 2D-QSAR and 3D-QSAR/CoMSIA studies on a series of (R)-2-((2-(1H-Indol-2-yl)ethyl)amino)-1-phenylethan-1-ol with human beta(3)-adrenergic activity. Molecules.

[25] Umar BA, Uzairu A, Shallangwa GA, Sani U (2019). QSAR modeling for the prediction of pGI50 activity of compounds on LOX IMVI cell line and ligand-based design of potent compounds using in silico virtual screening. Netw Model Anal Health Inform Bioinform.

[26] Poleboyina PK, Rampogu S, Doneti R, Pasha A, Poleboyina SM, Bhanothu S (2022). Screening and identification of potential inos inhibitors to curtail cervical cancer progression: an in silico drug repurposing approach. Appl Biochem Biotechnol.

[27] Vucicevic J, Nikolic K, Mitchell JBO (2019). rational drug design of antineoplastic agents using 3D-QSAR, cheminformatic, and virtual screening approaches. Curr Med Chem.

[28] Ibrahim MT, Uzairu A, Shallangwa GA, Uba S (2020). Structure-based design and activity modeling of novel epidermal growth factor receptor kinase inhibitors; an in silico approach. Scientific African.

[29] Abdizadeh T, Ghodsi R, Hadizadeh F (2017). 3D-QSAR (CoMFA, CoMSIA) and molecular docking studies on histone deacetylase 1 selective inhibitors. Recent Pat Anticancer Drug Discov.

[30] Vishwakarma K, Bhatt H (2021). Molecular modelling of quinoline derivatives as telomerase inhibitors through 3D-QSAR, molecular dynamics simulation, and molecular docking techniques. J Mol Model.

[31] Goudzal A, El Aissouq A, El Hamdani H, Hadaji EG, Ouammou A, Bouachrine M (2022). 3D-QSAR modeling and molecular docking studies on a series of 2, 4, 5-trisubstituted imidazole derivatives as CK2 inhibitors. J Biomol Struct Dyn.

[32] Aouidate A, Ghaleb A, Ghamali M, Chtita S, Ousaa A, Choukrad M (2018). Computer aided drug design based on 3D-QSAR and molecular docking studies of 5-(1H-indol-5-yl)-1,3,4-thiadiazol-2-amine derivatives as PIM2 inhibitors: a proposal to chemists. In Silico Pharmacol.

[33] Vavricka CJ, Li Q, Wu Y, Qi J, Wang M, Liu Y (2011). Structural and functional analysis of laninamivir and its octanoate prodrug reveals group specific mechanisms for influenza NA inhibition. PLoS Pathog.

[34] Aziz M, Ejaz SA, Tamam N, Siddique F, Riaz N, Qais FA (2022). Identification of potent inhibitors of NEK7 protein using a comprehensive computational approach. Sci Rep.

[35] Kar S, Roy K, Leszczynski J, Benfenati E (2022). In Silico tools and software to predict admet of new drug candidates. in silico methods for predicting drug toxicity.

[36] Babalola S, Igie N, Odeyemi I (2022) Structure-based discovery of multitarget directed anti-inflammatory p-nitrophenyl hydrazones; molecular docking, drug-likeness, in-silico pharmacokinetics, and toxicity studies

[37] Tropsha A (2010). Best practices for QSAR model development, validation, and exploitation. Mol Inf.

[38] Roy K, Das RN, Ambure P, Aher RB (2016). Be aware of error measures. Further studies on validation of predictive QSAR models. Chemom Intell Lab Syst.

[39] Roy K, Kar S, Das RN, Roy K, Kar S, Das RN (2015). Statistical methods in QSAR/QSPR. A primer on QSAR/QSPR modeling.

[40] Darnag R, Minaoui B, Fakir M (2017). QSAR models for prediction study of HIV protease inhibitors using support vector machines, neural networks and multiple linear regression. Arab J Chem.

[41] Thompson CG, Kim RS, Aloe AM, Becker BJ (2017). Extracting the variance inflation factor and other multicollinearity diagnostics from typical regression results. Basic Appl Soc Psychol.

[42] Wang T, Tang L, Luan F, Cordeiro M (2018). Prediction of the toxicity of binary mixtures by QSAR approach using the hypothetical descriptors. Int J Mol Sci.

[43] Shirvani P, Fassihi A (2021). In silico design of novel FAK inhibitors using integrated molecular docking, 3D-QSAR and molecular dynamics simulation studies. J Biomol Struct Dyn.

[44] Gu X, Wang Y, Wang M, Wang J, Li N (2021). Computational investigation of imidazopyridine analogs as protein kinase B (Akt1) allosteric inhibitors by using 3D-QSAR, molecular docking and molecular dynamics simulations. J Biomol Struct Dyn.

[45] Chauhan K, Singh P, Kumar V, Shukla PK, Siddiqi MI, Chauhan PM (2014). Investigation of Ugi-4CC derived 1H-tetrazol-5-yl-(aryl) methyl piperazinyl-6-fluoro-4-oxo-1,4-dihydroquinoline-3-carboxylic acid: synthesis, biology and 3D-QSAR analysis. Eur J Med Chem.

[46] Ahmed A, Saeed A, Ejaz SA, Aziz M, Hashmi MZ, Channar PA (2022). Novel adamantyl clubbed iminothiazolidinones as promising elastase inhibitors: design, synthesis, molecular docking, ADMET DFT studies. RSC Adv.

[47] Arámburo-Gálvez JG, Arvizu-Flores AA, Cárdenas-Torres FI, Cabrera-Chávez F, Ramírez-Torres GI, Flores-Mendoza LK (2022). Prediction of ACE-I inhibitory peptides derived from chickpea (*Cicer** arietinum* L.) in silico assessments using simulated enzymatic hydrolysis, molecular docking and ADMET evaluation. Foods.

[48] Adianingsih OR, Khasanah U, Anandhy KD, Yurina V (2022). In silico ADME-T and molecular docking study of phytoconstituents from Tithonia diversifolia (Hemsl.) A. Gray on various targets of diabetic nephropathy. J Pharm Pharmacogn Res.

[49] Dowdy SF, Setten RL, Cui X-S, Jadhav SG (2022). Delivery of RNA therapeutics: the great endosomal escape!. Nucleic Acid Therap.

[50] Hossen N, Hye T, Ahsan F (2022). Biopharmaceutics, pharmacokinetics, and pharmacodynamics of biological products. Biologics and biosimilars.

[51] Xu Y (2022). Deep neural networks for QSAR. Artificial intelligence in drug design.

[52] Xiong G, Wu Z, Yi J, Fu L, Yang Z, Hsieh C (2021). ADMETlab 2.0: an integrated online platform for accurate and comprehensive predictions of ADMET properties. Nucleic Acids Res.

